# Morpho-phylogenetic evidence uncovers new taxa of lignicolous freshwater Sordariomycetes from Yunnan and Guizhou provinces, China

**DOI:** 10.3897/mycokeys.128.180553

**Published:** 2026-02-05

**Authors:** Hong-Wei Shen, Dan-Feng Bao, Xing-Juan Xiao, Jayarama Darbhe Bhat, Yong-Zhong Lu, Zong-Long Luo

**Affiliations:** 1 College of Agriculture and Biological Science, Dali University, Dali 671003, Yunnan, China School of Science, Mae Fah Luang University Chiang Rai Thailand; 2 Co-Innovation Center for Cangshan Mountain and Erhai Lake Integrated Protection and Green Development of Yunnan Province, Dali University, Dali 671003, Yunnan, China Center of Excellence in Fungal Research, Mae Fah Luang University Chiang Rai Thailand; 3 Engineering Research Center of the Utilization for Characteristic Bio-Pharmaceutical Resources in Southwest, Ministry of Education, Guizhou University, Guiyang 550025, Guizhou, China Department of Botany and Microbiology, College of Science, King Saud University Riyadh Saudi Arabia; 4 School of Food and Pharmaceutical Engineering, Guizhou Institute of Technology, Guiyang 550025, Guizhou, China Ministry of Education, Guizhou University Guiyang China; 5 Center of Excellence in Fungal Research, Mae Fah Luang University, Chiang Rai 57100, Chiang Rai, Thailand College of Agriculture and Biological Science, Dali University Dali China; 6 School of Science, Mae Fah Luang University, Chiang Rai 57100, Chiang Rai, Thailand Co-Innovation Center for Cangshan Mountain and Erhai Lake Integrated Protection and Green Development of Yunnan Province, Dali University Dali China; 7 Department of Botany and Microbiology, College of Science, King Saud University, P.O. Box 2455, Riyadh 11451, Saudi Arabia School of Food and Pharmaceutical Engineering, Guizhou Institute of Technology Guiyang China

**Keywords:** Lignicolous freshwater fungi, Platytrachelaceae, taxonomy, Yunnan–Guizhou Plateau

## Abstract

Lignicolous freshwater fungi play an important role in nutrient cycling and the maintenance of biodiversity within freshwater ecosystems. Recent intensive studies, particularly in Asia, have greatly advanced understanding of their taxonomy, diversity, and ecology. Yunnan and Guizhou provinces in southwestern China represent one of the most active regions for freshwater fungal research due to their complex topography, diverse freshwater bodies, and rich biodiversity. As part of a comprehensive investigation of lignicolous freshwater fungi in southwestern China, specimens were collected from rivers and plateau lakes. Multi-gene phylogenetic analyses based on a combined LSU, ITS, SSU, *rpb*2, and *tef*1-α dataset revealed that four monotypic genera, *Aquimonospora*, *Multiseptisporium*, *Paradiplococcium*, and *Platytrachelon*, form a distinct clade within Diaporthomycetidae families *incertae sedis* and are closely related to Melanascomaceae, Neodictyosporiaceae, Papulosaceae, and Pseudostanjehughesiaceae. Accordingly, a new family, Platytrachelaceae, is proposed to accommodate these genera. In addition, the genus *Multiseptisporium* is established to accommodate the type species *M. aquaticum***sp. nov**., which produces sporidesmium-like conidia. This study highlights the phylogenetic diversity and morphological complexity of sporidesmium-like taxa and underscores the importance of integrating morphological and phylogenetic evidence to resolve their taxonomic relationships. The findings enrich the known diversity of lignicolous freshwater fungi in Yunnan and Guizhou and provide new insights into their taxonomy and ecological adaptation within freshwater ecosystems.

## Introduction

Lignicolous freshwater fungi are those colonizing woody substrates in a variety of freshwater habitats, including lentic habitats (such as lakes, ponds, and reservoirs), lotic habitats (such as rivers and streams), and artificial habitats (such as water-cooling towers) (Luo et al. 2004; Hyde et al. 2016; Calabon et al. 2022, 2023). As saprophytes, they play a crucial role in the decomposition of lignocellulosic material and the release of nutrients, thus contributing to the maintenance of biodiversity in freshwater ecosystems (Yuen et al. 1998; Bucher et al. 2004; Hyde et al. 2016). Lignicolous freshwater fungi have been the subject of extensive research globally and within Asia, particularly in China and Thailand, which have emerged as major centers of study (Zhang et al. 2011; Jones et al. 2014; Hyde et al. 2016, 2021; Su et al. 2016; Bao et al. 2018, 2019, 2020, 2021, 2023; Luo et al. 2018a, 2019; Dong et al. 2020, 2021a, b; Calabon et al. 2021, 2022, 2023; Shen et al. 2022a, b, 2024a; Fryar and Catcheside 2023; Fryar et al. 2023; Xu et al. 2023, 2024, 2025a; Yang et al. 2023; Wang et al. 2024a, b; Xiao et al. 2025). Significant advances in this field have been made through the concerted efforts of prominent research institutions such as the Center of Excellence in Fungal Research (Mae Fah Luang University), Chiang Mai University, the University of Hong Kong, Dali University, and the Kunming Institute of Botany, Chinese Academy of Sciences (KIB-CAS). Through the integration of detailed morphological characterization and multi-gene phylogenetic analyses, researchers have successfully discovered numerous novel species from diverse freshwater habitats (Su et al. 2016; Luo et al. 2019; Dong et al. 2020, 2021a; Calabon et al. 2021, 2022; Hyde et al. 2021; Bao et al. 2023; Yang et al. 2023; Shen et al. 2024a; Wang et al. 2024b). Among the regions contributing significantly to this progress, Yunnan and Guizhou provinces in southwestern China stand out as the most active and productive areas for freshwater fungal research, with numerous species documented from streams, rivers, and lakes (Cai et al. 2002; Luo et al. 2016, 2017, 2018a, b, 2019; Su et al. 2016; Bao et al. 2018, 2020, 2023; Dong et al. 2020, 2021b; Yang et al. 2021, 2023; Du et al. 2022; Shen et al. 2022a, b, 2023, 2024a, b; Shen and Luo 2023; Xu et al. 2023; Ma et al. 2024; Wang et al. 2024a, b; Liu et al. 2025).

Sordariomycetes is one of the largest and most ecologically diverse classes within the phylum Ascomycota, comprising numerous lineages that inhabit a wide range of terrestrial and aquatic ecosystems (Maharachchikumbura et al. 2015; Luo et al. 2019; Hyde et al. 2020, 2021; Huang et al. 2021; Bao et al. 2023). Among these, lignicolous freshwater fungi within Sordariomycetes represent a highly diverse and ecologically important group (Luo et al. 2019; Hyde et al. 2020, 2021; Bao et al. 2023; Yang et al. 2023). In recent years, intensive studies focusing on this group have greatly expanded our understanding of its taxonomic diversity, distribution patterns, and ecological significance in freshwater environments (Luo et al. 2019; Hyde et al. 2020, 2021; Calabon et al. 2021; Dong et al. 2021b; Bao et al. 2023; Yang et al. 2023). These investigations have led to the discovery and description of numerous new species, highlighting the hidden diversity of freshwater Sordariomycetes. Lignicolous freshwater species of Sordariomycetes are primarily assigned to orders such as Annulatascales, Chaetosphaeriales, Distoseptisporales, Hypocreales, Microascales, Pleurotheciales, Savoryellales, Sporidesmiales, and Diaporthomycetidae families *incertae sedis* (Luo et al. 2019; Réblová et al. 2020a, b; Dong et al. 2021a, b; Li et al. 2021; Yang et al. 2021, 2023; Calabon et al. 2022; Zhang et al. 2022; Bao et al. 2023; Yu et al. 2023; Wang et al. 2025b).

Many species within the class Sordariomycetes exhibit highly similar morphological characteristics, which pose significant challenges to traditional taxonomy that relies solely on morphology. Limited diagnostic features and overlapping characters often confound taxonomists, making it difficult to distinguish closely related taxa with sufficient confidence (Bhunjun et al. 2021; Jayawardena et al. 2021; Maharachchikumbura et al. 2021; Chen et al. 2023; Dissanayake et al. 2024). For example, some endophytic and pathogenic fungi, along with certain saprophytic taxa such as species of *Apiospora*, *Colletotrichum*, *Diaporthe*, *Fusarium*, chloridium-like taxa, and sporidesmium-like taxa, share remarkably similar morphological characteristics (Crous and Groenewald 2013; Su et al. 2016; Crous et al. 2021; Jayawardena et al. 2021; Koukol and Delgado 2021; Pintos and Alvarado 2021; Talhinhas and Baroncelli 2021; Monkai et al. 2022; Norphanphoun et al. 2022; Réblová et al. 2022; Réblová and Nekvindová 2023; Delgado et al. 2024; Dissanayake et al. 2024). These morphological similarities make it nearly impossible to achieve precise species delimitation through morphology alone (Bhunjun et al. 2021; Jayawardena et al. 2021; Maharachchikumbura et al. 2021; Chen et al. 2023; Dissanayake et al. 2024).

To overcome these limitations, the application of multi-gene phylogenetic analyses in conjunction with detailed morphological assessments has proven to be a powerful and indispensable approach. This integrative method has greatly enhanced the resolution of species boundaries and enabled the discovery of cryptic diversity within morphologically conserved groups (Bhunjun et al. 2021; Jayawardena et al. 2021; Maharachchikumbura et al. 2021; Chen et al. 2023; Dissanayake et al. 2024). Through comprehensive re-evaluation using both multi-gene phylogenetic analyses and morphological evidence, several underestimated or misclassified taxa have been correctly re-identified and newly described, contributing to a more accurate and refined classification framework for the group (Crous et al. 2021; Hyde et al. 2021; Jayawardena et al. 2021; Pintos and Alvarado 2021; Talhinhas and Baroncelli 2021; Réblová et al. 2022; Wu and Diao 2022; Bao et al. 2023; Réblová and Nekvindová 2023; Delgado et al. 2024; Dissanayake et al. 2024).

At present, we are undertaking a systematic and comprehensive investigation of the diversity and ecology of lignicolous freshwater fungi in southwestern China (Shen et al. 2022b, 2024a; Shen and Luo 2023; Wang et al. 2025a). These research programs encompass a wide array of freshwater habitats, including major rivers, tributaries, plateau lakes, and micro-water bodies in alpine regions. The sampling efforts span both internationally and nationally significant drainage basins, aiming to capture the ecological and taxonomic diversity of fungi associated with submerged woody substrates. In this study, lignicolous freshwater fungi were collected from a river and a plateau lake in Guizhou and Yunnan provinces. Through detailed morphological examinations combined with multi-gene phylogenetic analyses, three novel taxa were identified and are formally introduced in this paper. This study not only increases the known species richness of freshwater wood-inhabiting fungi in Yunnan but also enhances broader understanding of their distribution and phylogenetic relationships. The results underscore the importance of integrated taxonomic approaches in uncovering cryptic fungal diversity, particularly in underexplored and ecologically diverse regions such as international rivers, tributaries, plateau lakes, and micro-water bodies.

## Materials and methods

### Specimen collection, examination, and isolation

Specimens of submerged decaying wood were collected from Yilonghu Lake (Yunnan Province) in spring and from a stream (Guizhou Province) in autumn. The samples were brought to the laboratory and incubated in sterile, damp plastic boxes at room temperature for one week. Morphological observations were conducted following the methods of Shen et al. (2023). Macro-morphological characteristics of the samples were observed using an Optec SZ 760 compound stereomicroscope (Chongqing Optec Instrument Co., Ltd., Chongqing, China). Temporarily mounted microscope slides were examined and photographed under a Nikon ECLIPSE Ni-U compound stereomicroscope (Nikon, Tokyo, Japan). The morphological characteristics of colonies on their natural substrates were documented using a Nikon SMZ1000 stereo zoom microscope (Nikon, Tokyo, Japan). Photomicrograph measurements were performed using Tarosoft® Image Framework version 0.9.7. Image processing and layout adjustments were conducted using Adobe Photoshop CS5 Extended version 12.0.0.0 (Adobe Systems Inc., San Jose, CA, USA).

Single-spore isolations were carried out based on the method described by Shen et al. (2023). Germinated conidia were individually transferred to fresh potato dextrose agar plates (PDA; Beijing Bridge Technology Co., Ltd., Beijing, China) and incubated at ambient room temperature under alternating dark and light conditions to promote colony development and sporulation. To document the origin and development of germ tubes, germinated spores along with the surrounding agar were mounted in sterile water on glass slides and photographed under a microscope. Following microscopic observation and isolation, the fungal specimens were air-dried under ambient conditions, wrapped in absorbent paper, and sealed in zip-lock plastic bags with mothballs for long-term preservation. Voucher specimens were deposited in the Herbarium of the Kunming Institute of Botany, Chinese Academy of Sciences (**HKAS**), Kunming, China, and the Herbarium of the Guizhou Academy of Agricultural Sciences (**GZAAS**), Guiyang, China. Living cultures were deposited in the Kunming Institute of Botany Culture Collection (**KUNCC**), Kunming, China, and the Guizhou Culture Collection (**GZCC**), Guiyang, China. Fungal Names numbers were registered in the Fungal Names database (https://nmdc.cn/fungalnames/registe; accessed on 4 October 2025; Wang et al. 2023).

### DNA extraction, PCR amplification, and sequencing

DNA extraction, PCR amplification, sequencing, and phylogenetic analyses were carried out following the methods described by Dissanayake et al. (2020). Mycelia used for DNA extraction were cultivated on PDA for 3–4 weeks at 24 °C. From each isolate, total genomic DNA was extracted from 100–150 mg of axenic mycelium, which was carefully scraped from the edges of the growing culture with a sterile scalpel. The material was transferred to a 1.5 mL microcentrifuge tube using sterilized inoculum needles. Mycelium was ground to a fine powder with liquid nitrogen or quartz sand to disrupt the cells for DNA extraction. DNA was extracted using the Trelief^TM^ Plant Genomic DNA Kit (TSP101) following the manufacturer’s guidelines (Beijing Tsingke Biological Engineering Technology and Services Co., Ltd., Beijing, P.R. China).

Polymerase chain reaction (PCR) amplification was performed following the protocol described by Shen et al. (2024b), and the thermal cycling conditions are shown in Table 1. Five gene regions, internal transcribed spacer (ITS), large subunit ribosomal RNA (LSU), small subunit ribosomal RNA (SSU), translation elongation factor 1-alpha (*tef*1-α), and the second largest subunit of RNA polymerase II (*rpb*2), were amplified using the primer pairs ITS5/ITS4 (White et al. 1990), LR0R/LR7 (Vilgalys and Hester 1990), NS1/NS4 (White et al. 1990), EF1-983F/EF1-2218R (Liu et al. 1999), and RPB2-5F/RPB2-7cR (Liu et al. 1999), respectively. PCR products were purified using minicolumns, purification resin, and buffer according to the manufacturer’s protocols. Sequencing of purified PCR amplicons was performed by Beijing Tsingke Biological Engineering Technology and Services Co., Ltd. (Beijing, P.R. China).

**Table 1.** PCR thermocycling conditions for genes used in this paper.

Genes	Initial denaturation		Denaturation		Annealing		Extension		Final extension		No. of cycles
	Temp (°C)	Time (min)	Temp (°C)	Time (s)	Temp (°C)	Time (s)	Temp (°C)	Time (s)	Temp (°C)	Time (min)	
ITS, SSU	94	3	94	30	56	50	72	60	72	10	30
LSU	94	3	94	50	55	60	72	60	72	10	30
*tef*1-α	94	3	94	50	55	60	72	60	72	10	30
*rpb*2	94	5	94	60	52	90	72	90	72	10	38

### Phylogenetic analysis

BLAST searches were conducted using the BLASTn algorithm to retrieve closely related sequences from GenBank (http://www.ncbi.nlm.nih.gov). Multiple sequence alignments were performed using the MAFFT online service (version 7) with default settings (Kuraku et al. 2013; Katoh et al. 2019; http://mafft.cbrc.jp/alignment/server/index.html, accessed on 20 September 2025), and poorly aligned regions were trimmed using trimAl version 1.2 with default parameters (http://trimal.cgenomics.org for specific operation steps; Capella-Gutiérrez et al. 2009). Aligned sequences from different loci were concatenated using SequenceMatrix v1.7.8 (Vaidya et al. 2011). The resulting concatenated dataset in FASTA format was converted into PHYLIP and NEXUS formats using the ALignment Transformation EnviRonment (ALTER) online platform (http://sing.ei.uvigo.es/ALTER/, accessed on 20 September 2025).

Phylogenetic analyses were performed using both maximum likelihood (ML) and Bayesian inference (BI) approaches. ML analysis was performed using RAxML-HPC2 on XSEDE (8.2.12) (Stamatakis 2006; Stamatakis et al. 2008) in the CIPRES Science Gateway (Miller et al. 2010; http://www.phylo.org/portal2; accessed on 20 September 2025), applying the GTR+GAMMA model with 1,000 bootstrap replicates. Bayesian analysis was implemented in MrBayes version 3.2.6 (Ronquist et al. 2012), with the best-fit model of nucleotide substitution determined using MrModeltest 2.2 (Guindon and Gascuel 2003; Darriba et al. 2012). Posterior probabilities (PP) were estimated using a Markov chain Monte Carlo (MCMC) sampling approach (Rannala and Yang 1996). Six simultaneous Markov chains were run for 20,000,000 generations, with trees sampled every 1,000 generations, and the first 25% of trees discarded as burn-in.

Phylogenetic trees were visualized using FigTree v1.4.4 (http://tree.bio.ed.ac.uk/software/figtree) and subsequently edited and formatted for publication using Adobe Illustrator (Adobe Systems Inc., San Jose, CA, USA). Newly generated sequences were deposited in GenBank, and detailed strain information, including accession numbers and voucher data, is provided in Table 2. The alignments and phylogenetic trees were deposited in TreeBASE (http://www.treebase.org/, accession number 32456).

**Table 2.** Taxa used in the phylogenetic analyses and their corresponding GenBank accession numbers. Notes: The ex-type cultures are indicated using “T” after strain numbers; newly generated sequences are indicated in bold. A hyphen (–) stands for no sequence data in GenBank.

Taxon	Source	GenBank accession numbers				
		ITS	LSU	SSU	*rpb*2	*tef*1α
*Achroceratosphaeria potamia*	CBS 125414 ^T^	MH863679	GQ996538	GQ996541	KM588908	–
*Acrodictys bawanglingensis*	SAUCC 1342 ^T^	ON606324	ON614219	ON620164	ON859853	–
*Acrodictys chishuiensis*	GZCC 20-0513 ^T^	OP377810	OP377909	OP377995	OP473084	–
*Afroraffaelea ambrosiae*	CBS 141678 ^T^	OM632703	OM584293	–	OM631577	–
*Annulatascus tratensis*	MFLUCC 17-2055 ^T^	OP377891	OP377977	OP378052	–	–
*Annulatascus velatisporus*	MFLUCC 16-1441	KY320183	KY244031	KY244032	–	–
*Aquapteridospora hyalina*	GZCC 22-0072^T^	ON527937	ON527945	–	ON533689	ON533681
*Aquapteridospora jiangxiensis*	JAUCC 3008 ^T^	MZ871501	MZ871502	–	MZ855768	MZ855767
*Aquimonospora tratensis*	MFLUCC 17-2133 ^T^	MK335798	MK335797	MK335778	MK344654	–
*Ascolacicola aquatica*	CGMCC 3.27466	PQ644539	PQ650108	PQ844522	PQ824476	PQ824490
*Ascolacicola minispora*	KUNCC 23-14565 ^T^	OR589283	OR600931	OR743193	OR820903	OR739157
*Ascotaiwania lignicola*	NIL 00005	HQ446341	HQ446364	HQ446284	HQ446419	HQ446307
*Atractospora thailandensis*	KUMCC 16-0067 ^T^	MF374353	MF374362	MF374371	MF370951	MF370962
*Bactrodesmium diversum*	CBS 144081 ^T^	MN699356	MN699416	MN699372	MN704295	MN704320
*Bactrodesmium obovatum*	CBS 144077	MN699395	MN699424	MN699375	MN704298	MN704322
*Barbatosphaeria arboricola*	CBS 127689 ^T^	MH864681	MH876117	KM492849	KM492901	–
*Barbatosphaeria hippocrepida*	ICMP 17630 ^T^	KM492894	FJ617557	HQ878599	HQ878608	–
*Barbatosphaeria varioseptata*	CBS 137797 ^T^	KM492896	KM492869	KM492857	KM492907	–
*Barrmaelia macrospora*	CBS 142768 ^T^	KC774566	KC774566	–	MF488995	MF489005
*Barrmaelia moravica*	CBS 142769 ^T^	MF488987	MF488987	–	MF488996	MF489006
*Bullimyces communis*	AF281-5	–	JF775587	JF758619	–	–
*Cancellidium cinereum*	MFLUCC 18-0424 ^T^	MT370353	MT370363	MT370351	MT370486	MT370488
*Cancellidium griseonigrum*	MFLUCC 17-2117 ^T^	MT370354	MT370364	MT370352	MT370487	–
*Ceratolenta caudata*	CBS 125234 ^T^	–	JX066704	JX066708	JX066699	–
*Ceratosphaeria aquatica*	MFLUCC 18-1337 ^T^	MK828612	MK835812	–	MN156509	MN194065
*Ceratosphaeria lignicola*	MFLUCC 18-0342	MK828613	MK835813	–	–	MN194066
*Ceratostomella melanospora*	CBS 147993 ^T^	PQ215757	PQ215750	PQ215925	PQ213491	PQ213502
*Ceratostomella pyrenaica*	CBS 117116 ^T^	PQ215759	DQ076323	DQ076324	PQ213492	PQ213503
*Ceratostomella sordida*	CBS 116000	PQ215761	AY761087	AY761090	KY931929	PQ213505
*Chloridiopsiella preussii*	CBS 230.75 ^T^	OR134700	OR134645	–	OR135580	OR130780
*Chloridiopsis constrictospora*	CBS 432.92 ^T^	OR134704	OR134649	–	OR135584	OR130784
*Chlorociboria aeruginosa*	AFTOLID 151	DQ491501	AY544669	–	DQ470886	DQ471053
*Conlarium baiseense*	HMAS 247298 ^T^	MF083157	MF083158	MF083159	MK573000	–
*Conlarium nanningense*	HMAS 247075 ^T^	KX886204	KX886202	KX886203	MK572998	–
*Corollospora fusca*	NBRC 32107	JN943382	JN941483	JN941662	–	–
*Corollospora gracilis*	NBRC 32111	JN943386	JN941487	JN941659	–	–
*Dermea acerina*	AFTOLID 941	MH855942	DQ247801	DQ247809	DQ247791	DQ471091
*Diaporthostoma machili*	CFCC 52100 ^T^	MG682080	MG682020	–	MG682040	–
*Diaporthostoma machili*	CFCC 52101 ^T^	MG682081	MG682021	–	MG682041	–
*Diluviicola aquatica*	MFLUCC 15-0986 ^T^	MF374356	MF374365	–	MF370953	MF370961
*Distoseptispora bambusae*	MFLUCC 20-0091	MT232713	MT232718	MT232716	MT232881	MT232880
*Distoseptispora palmarum*	MFLUCC 18-1446	MK085062	MK079663	MK079661	MK087670	MK087660
*Entosordaria perfidiosa*	CBS 142773 ^T^	MF488993	MF488993	–	MF489003	MF489012
*Entosordaria quercina*	CBS 142774 ^T^	MF488994	MF488994	–	MF489004	MF489013
*Fluminicola aquatica*	MFLU 15-2710 ^T^	MF374357	MF374366	MF374374	–	MF370960
*Fluminicola saprophytica*	MFLUCC 15-0976 ^T^	MF374358	MF374367	MF374375	MF370954	MF370956
*Fluminicola striata*	MFLUCC 18-0990 ^T^	MW286496	MW287770	–	–	–
*Graphilbum crescericum*	CBS 130864 ^T^	OM501403	OM514749	–	OM631604	–
*Hawksworthiomyces crousii*	MUCL55928 ^T^	KX396551	KX396548	–	OM631609	–
*Jobellisia fraterna*	SMH 2863	–	AY346285	–	–	–
*Jobellisia guangdongensis*	HMAS 251240 ^T^	NR_138381	NG_068733	–	–	–
*Lentomitella magna*	ICMP 18371 ^T^	KY931786	KY931815	KY931871	KY931843	–
*Lindriella thalassiae*	AFTOL ID 413	DQ491508	DQ470947	DQ470994	FJ238382	DQ471065
*Lulworthia atlantica*	FCUL061107CP3	KT347208	JN886825	KT347196	–	OQ296422
*Lulworthia norwegica*	TRä3123A	OP683562	OP648294	OP680778	OP715904	OP715908
*Melanascoma panespora*	AD291710 ^T^	–	OQ789909	–	–	–
*Melanascoma panespora*	AD219607	–	OQ799385	–	–	OQ870569
*Myrmecridium hydei*	MFLUCC 23-0217 ^T^	OR500543	OR500545	–	OR515019	OR515020
*Neodictyosporium cheirosporum*	CGMCC 3.27450 ^T^	PQ067912	PQ067743	PQ066579	–	PQ271578
*Neodictyosporium cheirosporum*	UESTCC 24.0121 ^T^	PQ067906	PQ067737	PQ066573	PQ241075	PQ271577
*Neodictyosporium sexuale*	CGMCC 3.27441 ^T^	PQ067893	PQ067724	PQ066563	PQ241074	PQ271583
*Neodictyosporium sexuale*	UESTCC 24.0112 ^T^	PQ067892	PQ067723	PQ066562	PQ241073	–
*Neomyrmecridium aquaticum*	S-1158	MK828656	MK849803	MK828322	MN124540	MN194061
*Nigroconidius bambusae*	CGMCC 3.27446 ^T^	PQ067898	PQ067729	PQ066566	PQ247602	PQ280792
*Nigroconidius bambusae*	UESTCC 24.0160	PQ067909	PQ067739	PQ066575	PQ247603	PQ280793
*Nigroconidius septatus*	CGMCC 3.27449 ^T^	PQ067905	PQ067736	PQ066572	PQ247605	PQ280797
*Nigroconidius septatus*	UESTCC 24.0162	PQ067904	PQ067733	PQ066570	PQ247606	PQ280795
*Ophioceras dolichostomum*	CBS 114926	JX134677	JX134689	JX134663	–	JX134703
*Ophioceras thailandense*	MFLUCC 15-0603 ^T^	OP377882	OP377968	OP378045	–	OP473057
*Papulosa amerospora*	AFTOL ID 748	–	DQ470950	DQ470998	DQ470901	DQ471069
*Paradiplococcium singulare*	FMR 10752 ^T^	KY853456	KY853517	–	–	–
*Paralulworthia gigaspora*	MUT 5086	MZ357716	MZ357738	MZ357759	MZ357221	MZ407522
*Paraproliferophorum hyphaenes*	CPC 40103 ^T^	ON603770	ON603790	–	–	ON605632
*Pararamichloridium caricicola*	CBS 145069 ^T^	MK047438	MK047488	–	–	–
*Pararamichloridium verrucosa*	CBS 128.86 ^T^	MH861933	MH873621	NG_065540	–	–
***Multiseptisporium aquaticum***	**KUNCC 23-14304 ^T^**	**PX230562**	**PX230548**	**–**	**PX238393**	**PX238386**
***Multiseptisporium aquaticum***	**GZCC 24-0036**	**PX230563**	**PX230549**	**PX230534**	**PX238394**	**PX238387**
*Perennicordyceps cuboidea*	NBRC 103836	JN943332	JN941420	JN941721	AB972955	AB972951
*Perennicordyceps paracuboidea*	NBRC 100942	JN943337	JN941430	JN941711	AB972958	AB972954
*Phaeoacremonium novae-zealandiae*	CBS 110156 ^T^	MH862854	NG_068987	NG_062040	–	–
*Phaeoacremonium rubrigenum*	CBS 498.94 ^T^	MH862477	MH874125	–	–	–
*Phomatospora bellaminuta*	AFTOLID 766	–	FJ176857	–	FJ238345	–
*Phomatospora uniseriata*	MFLUCC 17-2068 ^T^	MT310660	MT214616	MT226727	MT394720	MT394672
*Pisorisporium cymbiforme*	PRM 924378	–	KM588902	KM588899	KM588905	–
*Pisorisporium cymbiforme*	PRM 924379	–	KM588903	KM588900	KM588906	–
*Pisorisporium cymbiforme*	CBS 138884	–	KM588904	KM588901	KM588907	–
*Platytrachelon abietis*	CBS 125235 ^T^	–	NG_057957	NG_061135	JX066698	–
*Pleurostoma ootheca*	CBS 115329 ^T^	HQ878590	AY761079	AY761074	HQ878606	–
*Pleurostoma richardsiae*	CBS 270.33 ^T^	AY179948	AB364684	AY761066	HQ878607	–
*Proliferophorum thailandicum*	MFLUCC 17-0293	MK028344	MK028343	MK028345	–	–
*Pseudodactylaria longidenticulata*	MFLUCC 17-2383 ^T^	OP377857	OP377942	OP378023	OP473102	OP473036
*Pseudodactylaria uniseptata*	MFLUCC 17-2395 ^T^	OP377892	OP377978	OP378053	OP473122	OP473063
*Pseudohalonectria aurantiaca*	MFLUCC 15-0379 ^T^	OP377881	OP377967	OP378044	–	OP473056
*Pseudohalonectria hampshirensis*	isolate 168	–	KX426220	KX426222	–	KX426225
*Pseudohalonectria hampshirensis*	isolate 177	–	KX426218	KX426221	–	KX426224
*Pseudomelanconis caryae*	CFCC 52110 ^T^	MG682082	NG_067555	–	MG682042	–
*Pseudomelanconis caryae*	CFCC 52112 ^T^	MG682084	MG682024	–	MG682044	–
*Pseudoplagiostoma alsophilae*	SAUCC WZ0451 ^T^	OP810625	OP810631	–	OP828578	–
*Pseudoplagiostoma altingiae*	CGMCC 3.28244 ^T^	PQ338993	PQ339638	–	PQ367345	–
*Pseudoproboscispora thailandensis*	MFLUCC 15-0989 ^T^	MF374360	MF374369	MF374377	–	MF370959
*Pseudostanjehughesia aquitropica*	MFLUCC 16-0569 ^T^	MF077548	MF077559	MF077537	–	MF135655
*Pseudostanjehughesia hydei*	KUNCC 23-14302	PQ845936	PX245755	PX218427	PX233791	PX238285
*Pseudostanjehughesia hydei*	KUNCC 23-14310 ^T^	PQ845937	PX245756	PX218428	PX233792	PX238286
*Pseudostanjehughesia lignicola*	MFLUCC 15-0352 ^T^	MK828643	MK849787	–	MN124534	MN194047
*Pseudostanjehughesia nielamuensis*	KUNCC 10407 ^T^	OP626330	PQ152658	PQ218305	PV657977	PV525047
*Pseudostanjehughesia nielamuensis*	KUNCC 24-18109	PQ168269	PQ152659	PQ218306	PV657978	PV525048
*Pseudothailandiomyces nypae*	MFLUCC 24-019 ^T^	–	PP621108	PP639237	PP780199	PP761025
*Rhamphoria delicatula*	MR1396	MG600390	AF261068	AF242267	KT991655	–
*Rhamphoriopsis aquimicrospora*	GZCC 20-0515 ^T^	OP377812	OP377911	OP377996	OP473085	OP472992
*Rhodoveronaea aquatica*	GZCC 20-0447	OP377862	OP377947	OP378027	OP473107	OP473041
*Rubellisphaeria abscondita*	CBS 132078 ^T^	KT991678	KT991666	KT991646	KT991657	–
*Spadicoides hyalostoma*	CBS 131268	KY931799	KY931827	KY931884	KY931854	–
*Sporidesmiella hyalosperma*	KUMCC 150431	MK828690	MK849841	MK828306	MN124522	MN194032
*Sporidesmiella novae-zelandiae*	S1256	MK828693	MK849845	–	MN124525	MN194036
*Sporidesmium aquaticivaginatum*	MFLUCC 15-0624 ^T^	KX710147	KX710142	MF077541	MF135647	MF135660
*Sporidesmium guizhouense*	CGMCC 3.19605 ^T^	MK818587	MK818586	MK818588	MK828516	MK828512
*Sporidesmium olivaceoconidium*	MFLUCC 15-0380 ^T^	KX710144	KX710139	MF077542	MF135648	MF135661
*Thailandiomyces bisetulosus*	BCC 00018	–	EF622230	EF622229	–	–
*Thyridium flavostromatum*	MAFF 247509 ^T^	LC655959	LC655963	–	LC655967	LC655971
*Thyridium punctulatum*	MAFF 247510 ^T^	LC655962	LC655966	–	LC655970	LC655974
*Torrentispora aquatica*	HKU(M) 17484	–	AY780365	–	–	–
*Torrentispora fibrosa*	ICMP 15147	KY931806	EF577060	KY931890	KY931859	–
*Wongia aquatica*	MFLUCC 18-1607 ^T^	MK828645	MK849788	MK828312	MN124536	MN194048
*Wongia flava*	CGMCC 3.25434 ^T^	OR589341	OR769700	OR743229	OR820915	OR739187
*Wongia miscanthi*	BCRC FU32062 ^T^	LC822730	LC822732	LC822736	LC822734	LC822738
*Wongia suae*	CGMCC 3.24295 ^T^	OQ911478	OQ911483	OQ998925	OR039047	OR039046
*Woswasia atropurpurea*	WU32007 ^T^	NR_154480	JX233658	JX233658	JX233659	–
*Xenodactylaria thailandica*	CBS 145074 ^T^	MK047443	MK047493	–	–	–
*Xylochrysis lucida*	CBS 135996 ^T^	NR_132063	KF539911	KF539912	KF539913	–

Notes: The ex-type cultures are indicated using “T” after strain numbers; newly generated sequences are indicated in bold. A hyphen (–) stands for no sequence data in GenBank.

## Results

### Phylogenetic analysis

Maximum likelihood (ML) phylogenetic analysis was conducted using a concatenated dataset of LSU, SSU, *rpb*2, and *tef*1-α sequences (4,056 characters, including gaps) from 129 ingroup strains, primarily within Diaporthomycetidae. The analysis resolved a monophyletic clade comprising four monotypic genera, which clustered with Papulosaceae within Diaporthomycetidae families *incertae sedis* with 62% ML bootstrap support (Suppl. material 1: fig. S1). This clade includes *Aquimonospora tratensis* (MFLUCC 17-2133), *Multiseptisporium aquaticum* gen. et sp. nov. (KUNCC 23-14304 and GZCC 24-0036), *Paradiplococcium singulare* (FMR 10752), and *Platytrachelon abietis* (CBS 125235).

**Figure 1.**
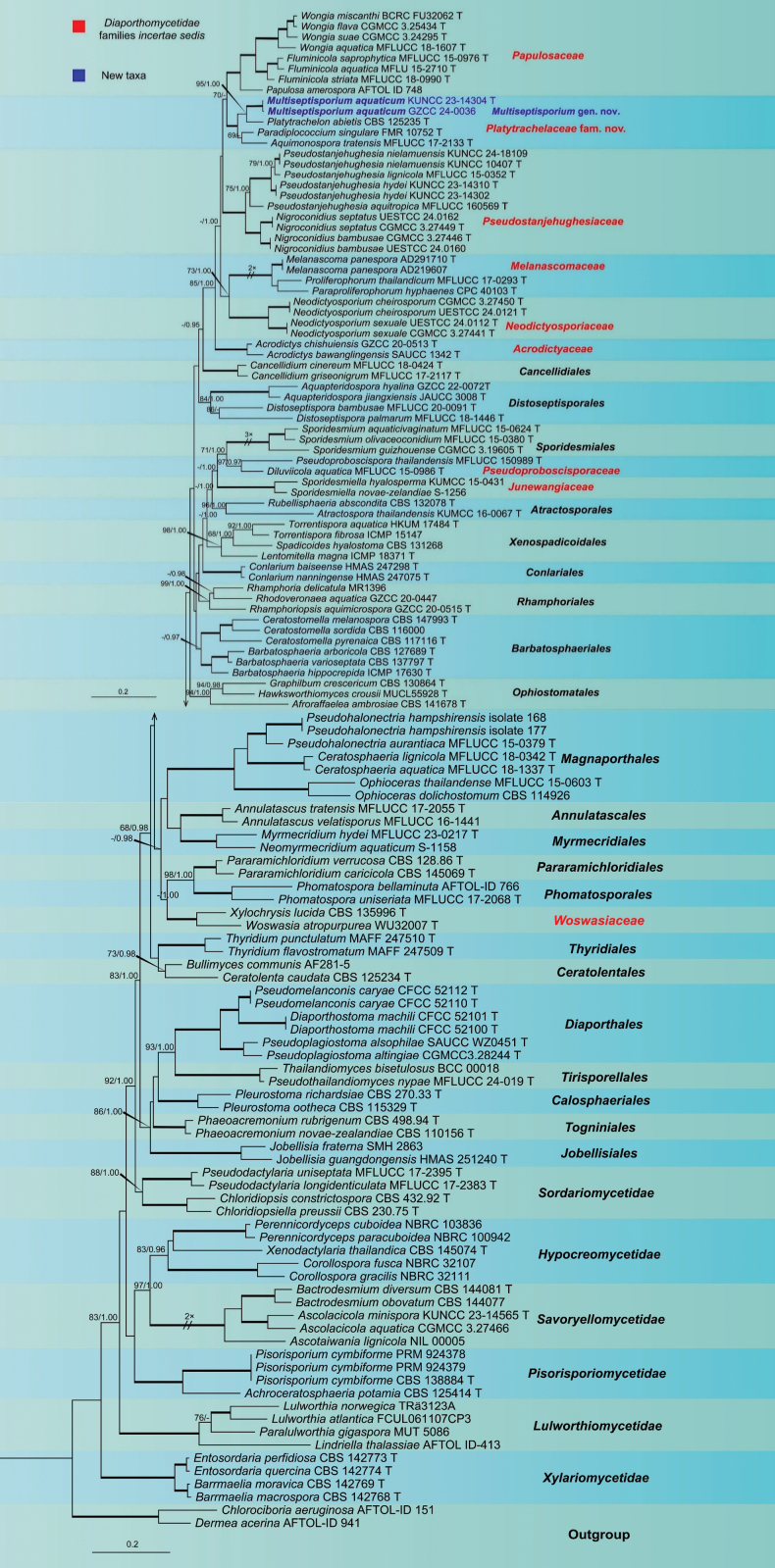
Phylogram generated from maximum likelihood analysis based on a combined LSU, ITS, SSU, *rpb*2, and *tef*1-α sequence dataset representing Sordariomycetes. A total of 129 strains are included in the combined analyses, comprising 4,056 characters, including gaps (753 characters for LSU, 491 characters for ITS, 1,003 characters for SSU, 953 characters for *rpb*2, and 856 characters for *tef*1-α). *Chlorociboria aeruginosa* (AFTOL-ID 151) and *Dermea acerina* (AFTOL-ID 941) were selected as the outgroup taxa. Phylogenetic trees generated from ML and BI analyses were similar in overall topology. The likelihood of the final tree was evaluated and optimized under GAMMA. The best RAxML tree with a final likelihood value of −99,500.004295 is presented. The matrix contained 2,581 distinct alignment patterns, with 26.92% undetermined characters or gaps. Estimated base frequencies were as follows: A = 0.242196, C = 0.257588, G = 0.279259, T = 0.220957; substitution rates AC = 1.228123, AG = 2.867394, AT = 1.190549, CG = 1.260961, CT = 6.459408, GT = 1.000000; α = 0.344777; tree length = 16.144614. Bayesian analyses generated 3,492 trees (average standard deviation of split frequencies = 0.009982), from which 2,620 trees were sampled after 25% were discarded as burn-in. The alignment contained a total of 2,581 unique site patterns. Bootstrap support values with ML ≥ 65% and Bayesian posterior probabilities (PP) ≥ 0.96 are given above the nodes as MLBS/BYPP. Thickened branches represent nodes with MLBS values of 100% and BYPP values of 1.00. A hyphen (–) indicates values lower than 65% MLBS or 0.95 PP. The ex-type cultures are indicated using “T” after strain numbers, and newly generated sequences are shown in bold blue.

A second ML analysis based on an expanded dataset combining LSU, ITS, SSU, *rpb*2, and *tef*1-α sequences (4,056 characters, including gaps) produced a similar topology, with the same four taxa forming a monophyletic lineage sister to Papulosaceae (Fig. 1). Phylogenetic trees inferred from ML and Bayesian inference (BI) analyses were largely congruent in overall topology (Fig. 1), with *Chlorociboria aeruginosa* (AFTOL-ID 151) and *Dermea acerina* (AFTOL-ID 941) selected as outgroup taxa. In addition, phylogenetic analyses based on individual gene regions were conducted and are presented in the Supplementary Materials (Suppl. material 1: figs S2–S6).

### Taxonomy

#### Platytrachelaceae

Taxon classificationFungiSordariomycetesPlatytrachelaceae

H.W. Shen & Z.L. Luo fam. nov.

352BFA77-33B7-5926-9429-F6EB088D4552

Fungal Names: FN 573256

##### Etymology.

The family name Platytrachelaceae is derived from the type genus *Platytrachelon*.

##### Type genus.

*Platytrachelon* Réblová, detailed description and illustrations by Réblová (2013).

##### Description.

***Saprobic*** on decaying wood. **Asexual morph**: Hyphomycetous. ***Mycelium*** immersed to superficial, composed of pale brown to brown, septate, branched hyphae. ***Conidiophores*** semi-macronematous to macronematous, mononematous, cylindrical, septate, unbranched, or reduced to conidiogenous cells. ***Conidiogenous cells*** enteroblastic, mono- to polyblastic, phialidic, polytretic, integrated or discrete, terminal or intercalary, cylindrical, clavate, ampulliform, spherical, sympodial, with denticulate or collarette, proliferating or non-proliferating. ***Conidia*** solitary or in short chains, hyaline to dark brown, ellipsoidal, obovoid to cylindrical, obclavate to rostrate, falcate or filiform, aseptate to multi-septate, with or without a sheath and appendages. **Sexual morph**: ***Ascomata*** immersed to superficial, subglobose to globose, dark brown to black, uniloculate. ***Necks*** central, cylindrical, ostiolate. ***Peridium*** leathery, two-layered. ***Paraphyses*** hyaline, branched, septate. ***Asci*** 8-spored, unitunicate, cylindric-clavate, long pedicellate, with an apical annulus. ***Ascospores*** uniseriate, hyaline, fusiform, septate.

##### Accepted genera.

*Aquimonospora*, *Multiseptisporium*, *Paradiplococcium*, and *Platytrachelon*.

##### Notes.

The new family Platytrachelaceae is proposed to accommodate *Multiseptisporium* gen. nov. and three previously monotypic genera, *Aquimonospora*, *Paradiplococcium*, and *Platytrachelon*, which were formerly placed in Diaporthomycetidae genera incertae sedis and Sordariomycetes genera *incertae sedis* (Hyde et al. 2024). In our multi-gene phylogenetic analyses, these four genera consistently formed an independent clade, clearly distinct from all currently recognized families within Diaporthomycetidae families *incertae sedis* (Fig. 1, Suppl. material 1: fig. S1). Consistent results were also recovered in single-gene maximum likelihood analyses, in which Platytrachelaceae repeatedly emerged as an independent lineage distinct from other families and orders within Diaporthomycetidae (Suppl. material 1: figs S2–S6). However, due to relatively low support values for the backbone of this lineage, we tentatively place it in Diaporthomycetidae families *incertae sedis*. The family is typified by the genus *Platytrachelon*, which was introduced by Réblová (2013) to accommodate *P. abietis* (formerly *Ceratosphaeria abietis*), based on a combination of morphological characteristics and phylogenetic placement. *Platytrachelon abietis* was originally described based on its sexual morph, and subsequent studies documented its asexual morph on PCA medium, including the presence of synanamorphs (Réblová 2013). Although *Platytrachelon* remains monotypic, it is currently the only genus within Platytrachelaceae for which both sexual and asexual morphs have been documented. This morphological completeness supports the designation of *Platytrachelon* as the type genus of Platytrachelaceae.

#### Multiseptisporium

Taxon classificationFungiSordariomycetesPlatytrachelaceae

H.W. Shen & Z.L. Luo gen. nov.

F65D5EFF-9BB8-52BA-BE14-3E8C69F1B831

Fungal Names: FN 573257

##### Etymology.

From Latin multi- (many), septum (a partition or septum), and sporium (spore), referring to the multi-septate conidia of the genus.

##### Type species.

*Multiseptisporium aquaticum* H.W. Shen & Z.L. Luo, sp. nov.

##### Description.

***Saprobic*** on submerged decaying wood. **Sexual morph**: Undetermined. **Asexual morph**: ***Colonies*** on natural substrate, effuse, scattered, superficial, hairy, dark brown. ***Mycelium*** partly immersed and partly superficial. ***Conidiophores*** macronematous, mononematous, cylindrical, septate, unbranched. ***Conidiogenous cells*** monoblastic, integrated, terminal, cylindrical, sometimes percurrently proliferating. ***Conidial secession*** rhexolytic. ***Conidia*** acrogenous, solitary, obclavate to rostrate, truncated at the base, brown to dark brown, euseptate, guttulate, smooth, occasionally verruculous, with a light-colored, truncated, cuneiform basal cell and a subhyaline, acuminate apical cell, with or without sheath and appendages.

##### Notes.

Multi-gene phylogenetic analyses revealed that the two collections of *Multiseptisporium* formed a distinct monophyletic clade that clustered with three monotypic genera, *Aquimonospora*, *Paradiplococcium*, and *Platytrachelon*, within Diaporthomycetidae genera incertae sedis and Sordariomycetes genera *incertae sedis* (Hyde et al. 2024; Fig. 1, Suppl. material 1: fig. S1). *Multiseptisporium* from these genera by producing a sporidesmium-like asexual morph, whereas its sexual morph remains undetermined. In contrast, *Aquimonospora* produces clavate, obovoid, or fusiform, septate conidia arising from integrated, terminal, monoblastic, enteroblastic conidiogenous cells (Yang et al. 2019). *Paradiplococcium* is characterized by subcylindrical to obovoid conidia in short chains and derived from polytretic, integrated or discrete, terminal or intercalary conidiogenous cells (Hernández-Restrepo et al. 2011). In culture, *Paradiplococcium* also produces a synanamorph with hyaline, falcate to filiform conidia developed from polyblastic, sympodial, denticulate conidiogenous cells (Hernández-Restrepo et al. 2011). *Platytrachelon* is known to produce both sexual and asexual morphs. Its asexual morph has been observed on PCA medium, where ellipsoidal, septate conidia are produced either directly from hyphae or from blastic, sympodial conidiogenous cells (Réblová 2013). Additionally, synanamorphic conidia are produced from blastic, phialidic conidiogenous cells. The clear morphological distinctions among these genera, combined with their phylogenetic relationships, support the recognition of *Multiseptisporium* as a novel genus.

#### Multiseptisporium aquaticum

Taxon classificationFungiSordariomycetesPlatytrachelaceae

H.W. Shen & Z.L. Luo sp. nov.

63F12CA9-E5EB-5714-949F-A72EE53AFF0C

Fungal Names: FN 573258

**Figure 2.**
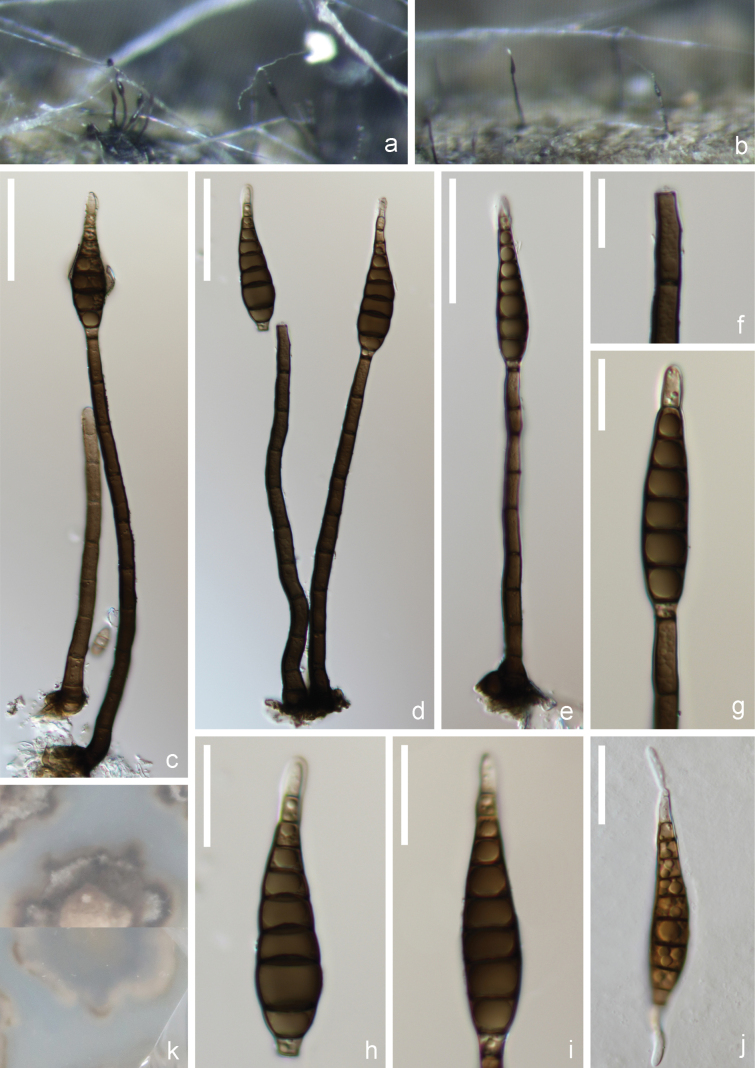
*Multiseptisporium aquaticum* (HKAS 151655, holotype). **a, b**. Colonies on woody substrates; **c–e**. Conidiophores with conidia; **f**. Conidiogenous cell; **g**. Conidiogenous cell with conidia; **h, i**. Conidia; **j**. Germinated conidium; **k**. Culture on PDA, upper surface and reverse. Scale bars: 40 μm (**c–e**); 15 μm (**f, g**); 20 μm (**h–j**).

Fig. 2 Chinese name: 水生多隔孢 (Pinyin: ShuĬ shēng duō gé bāo)

##### Etymology.

The specific epithet “*aquaticum*” refers to the aquatic habitat where the fungus was collected.

##### Holotype.

HKAS 151655.

##### Description.

***Saprobic*** on submerged decaying wood in freshwater habitats. **Sexual morph**: Undetermined. **Asexual morph**: ***Colonies*** on natural substrate, superficial, effuse, scattered, hairy, dark brown, solitary, or in small groups of 2–3. ***Mycelium*** partly immersed and partly superficial, composed of branched, septate, brown to dark brown, smooth-walled hyphae. ***Conidiophores*** (104–)120–204(–234) × 6–8 µm (x– = 162 × 7 µm, SD = 41.6 × 0.6, n = 15), macronematous, mononematous, cylindrical, straight or slightly flexuous, unbranched, 8–9-septate, slightly enlarged at the base, dark brown to black, smooth-walled. ***Conidiogenous cells*** (11–)14–21(–23) × 4–6 µm (x– = 18 × 5 µm, SD = 3.0 × 0.5, n = 15), monoblastic, integrated, terminal, determinate, cylindrical, slightly narrow at the top, brown, smooth-walled. ***Conidial secession*** rhexolytic. ***Conidia*** (53–)57–68(–75) × 10–14 µm (x– = 63 × 12 µm, SD = 5.7 × 1.2, n = 15), acrogenous, solitary, straight to slightly curved, 8–10-euseptate, obclavate to rostrate, truncated at base, tapering towards an acuminate apex, brown to dark olivaceous brown, subhyaline at apex, typically widest at third cell from base, with cuneiform basal cell, similar in color to apical cells, slightly constricted at septa, guttulate, smooth, occasionally verruculous, sometimes with an inconspicuous sheath at apex.

##### Culture characteristics.

Conidia germinating on PDA within 12 h and germ tubes produced from both ends. Colonies on PDA reaching 2–3 cm in diameter after two months at room temperature (around 22 °C) in the dark, dense, velvety, gray to dark brown at the margins, pale to gray at the center, with irregular margins and bulge center from above; dense, gray to dark gray, with irregular margins from below.

##### Material examined.

China • Yunnan Province, Honghe Hani and Yi Autonomous Prefecture, Shiping County, Yilonghu Lake, 23°40'56.4"N, 101°43'46.8"E, elevation 1400 m, on the unknown submerged decaying wood, 23 February 2023, H.W. Shen, L2194 (HKAS 151655, holotype), ex-type KUNCC 23-14304; • ibid. Guizhou Province, Zunyi City, Xishui County, 28°9'17"N, 105°55'50"E, elevation 640 m, on the unknown submerged decaying wood, 2 October 2023, X.J. Xiao, NJS15 (HKAS 152855, paratype), ex-paratype GZCC 24-0036.

##### Notes.

Phylogenetic analysis revealed that *Multiseptisporium aquaticum* (KUNCC 23-14304 and GZCC 24-0036) clustered with *Platytrachelon abietis* (CBS 125235) with stable support (Fig. 1). *Platytrachelon abietis*, originally described as *Ceratosphaeria abietis*, was collected from the inner side of decaying bark of *Abies alba* in the Czech Republic and has also been reported from Ukraine (Réblová 2013). Based on morphological and phylogenetic evidence, Réblová (2013) transferred *C. abietis* to the newly established genus *Platytrachelon*. Both sexual and asexual morphs of *P. abietis* have been documented. Notably, the asexual morph, including a synanamorph, was observed on PCA culture media. The asexual form of *P. abietis* is characterized by spherical, hyaline to subhyaline, holoblastic conidiogenous cells that proliferate sympodially; ellipsoidal, septate, smooth-walled conidia, sometimes arising directly from subhyaline or pigmented hyphae. The synanamorph is characterized by cylindrical to ampulliform, one-celled, hyaline to subhyaline conidiogenous cells terminating in a single phialidic opening or with an additional lateral opening; the collarette is shallow, wedge-shaped, and inconspicuous; conidia are cylindrical, strongly curved, hyaline, one-celled, and smooth-walled (Réblová 2013). *Multiseptisporium aquaticum* differs from *P. abietis* in having sporidesmium-like conidia produced from monoblastic, terminal, cylindrical conidiogenous cells, with rhexolytic conidial secession. No synanamorphs were observed. Comparison of nucleotide base differences in LSU and *rpb*2 sequence data between *M. aquaticum* (KUNCC 23-14304) and *P. abietis* (CBS 125235) showed differences of 3.2% (34/1,071 bp, including four gaps) and 11.8% (122/1,032 bp), respectively. Based on the combined morphological characteristics and sequence data, *M. aquaticum* is proposed as a new species.

## Discussion

Phylogenetic analyses revealed that four monotypic genera, *Aquimonospora*, *Multiseptisporium*, *Paradiplococcium*, and *Platytrachelon*, clustered within a well-supported monophyletic lineage related to Papulosaceae, Pseudostanjehughesiaceae, Melanascomaceae, and Neodictyosporiaceae in Diaporthomycetidae families *incertae sedis* (Hyde et al. 2024; Yu et al. 2024; Fig. 1, Suppl. material 1: fig. S1). The earliest described species within this lineage, *Platytrachelon abietis*, was initially assigned to Papulosaceae based on morphological and phylogenetic evidence (Réblová 2013). However, subsequent studies indicated that it does not belong to Papulosaceae, and it has since been treated as a member of Diaporthomycetidae genera *incertae sedis* (Réblová et al. 2016, 2018; Luo et al. 2019; Yang et al. 2019; Hyde et al. 2024). Based on morphological distinctiveness, such as discrete conidiogenous cells and short chains of conidia, and phylogenetic analyses of combined LSU and SSU sequences, *Paradiplococcium singulare*, originally described as *Diplococcium singulare* and later transferred to the monotypic genus *Paradiplococcium* by Hernandez-Restrepo et al. (2017), was subsequently placed in Sordariomycetes incertae sedis. *Aquimonospora tratensis* was established by Yang et al. (2019) from submerged woody substrates in freshwater habitats in Thailand. Its conidia are clavate, obovoid, or fusiform and composed of two parts: an upper part with brown to dark brown conidial cells and a lower part of two subhyaline to pale brown cells. Phylogenetic analyses showed that *A. tratensis* is closely related to *P. abietis* and belongs to the Diaporthomycetidae genera *incertae sedis* (Yang et al. 2019). Our multi-gene phylogenetic analyses, based on combined LSU, ITS, SSU, *rpb*2, and *tef*1-α datasets, yielded results consistent with those of Yang et al. (2019), showing that these four monotypic genera form a distinct and strongly supported clade related to Papulosaceae. Based on these results, a new family, Platytrachelaceae, is proposed to accommodate these four genera within the Diaporthomycetidae families *incertae sedis*.

*Paradiplococcium singulare* and *P. abietis* have been examined from both natural materials and artificial cultures, where they produced asexual morphs and synanamorphs on PCA and OA media (Réblová 2013; Hernandez-Restrepo et al. 2017). In contrast, *A. tratensis* and *M. aquaticum* are known only from natural substrates, with no asexual morphs observed in culture. Notably, *Pa. singulare* and *P. abietis* readily sporulate on PCA and OA media, suggesting that these media may promote conidial development in this lineage and related taxa (Réblová 2013; Hernandez-Restrepo et al. 2017; Crous et al. 2019; Réblová and Nekvindová 2023). We plan to culture our isolates on PCA and OA media to induce sporulation and obtain additional morphological characteristics that may provide further insights into their taxonomy (Su et al. 2012; Liao et al. 2025). The induction of sporulation in culture and the subsequent characterization of cultural morphs represent essential approaches for studying fungal diversity. Such methods are not only valuable for endophytic fungi but also play a crucial role in the investigation of saprobic taxa (Crous et al. 2019; Réblová et al. 2021; Réblová and Nekvindová 2023; Liao et al. 2025). Compared with morphological features observed on natural substrates, sporulation in culture often reveals diagnostic characteristics that are consistent with those on natural substrates and are critical for accurate species and genus delimitation (Réblová 2013; Hernandez-Restrepo et al. 2017).

Sporidesmium-like taxa are phylogenetically diverse, occurring predominantly in Sordariomycetes and Dothideomycetes, with a few representatives also reported from Leotiomycetes (Shenoy et al. 2006; Su et al. 2016; Liu et al. 2024). This group is morphologically complex, exhibiting a wide range of conidiophore structures—solitary or fasciculate, dematiaceous, septate, branched or unbranched—and often percurrently proliferating from cut ends; conidiogenous cells mono- or polyblastic; and conidia obclavate to rostrate, eu- to distoseptate, with or without gelatinous sheaths or appendages (Shenoy et al. 2006; Su et al. 2016; Zhang et al. 2022; Delgado et al. 2024). However, these morphological features have proven phylogenetically unreliable for generic delimitation, and in the absence of molecular data, species assignment based solely on morphology remains problematic (Shenoy et al. 2006; Su et al. 2016; Zhang et al. 2022; Delgado et al. 2024). With the increasing application of culture-dependent studies and multi-gene phylogenetic analyses, more robust insights into the classification and evolution of sporidesmium-like taxa have been achieved (Su et al. 2016; Wu and Diao 2022; Zhang et al. 2022; Delgado et al. 2024; Tang et al. 2024). In the present study, a new sporidesmium-like genus, *Multiseptisporium*, is introduced to accommodate a novel freshwater species, *M. aquaticum*, collected from China. Morphologically, *M. aquaticum* resembles *Sporidesmium* species in having macronematous, mononematous, cylindrical, septate conidiophores; monoblastic, cylindrical, integrated conidiogenous cells; and acrogenous, solitary, obclavate to rostrate, euseptate, guttulate conidia with or without a sheath (Shenoy et al. 2006; Su et al. 2016; Liu et al. 2024). However, phylogenetic analyses based on combined LSU, ITS, SSU, *rpb*2, and *tef*1-α datasets clearly separate *M. aquaticum* from other sporidesmium-like genera within Sordariomycetes, such as *Distoseptispora* (Distoseptisporales) and *Sporidesmium* (Sporidesmiales) (Fig. 1, Suppl. material 1: fig. S1). Accordingly, *Multiseptisporium* is proposed as a new genus based on its distinct phylogenetic position.

Species within Platytrachelaceae appear to be relatively rare, as all four currently recognized genera are monotypic and exhibit distinct ecological preferences and geographical distributions. *A. tratensis* is known solely from a single collection on submerged decaying wood in a freshwater habitat in Thailand, suggesting a narrow ecological amplitude and possible specialization to tropical freshwater woody substrates (Yang et al. 2019). *Multiseptisporium aquaticum* has been recorded from submerged woody substrates in a lake in Yunnan Province and a stream in Guizhou Province, China, indicating an aquatic lignicolous lifestyle with a degree of ecological flexibility. In contrast, *Pa. singulare* was collected from decaying wood of unknown origin in Spain, whereas *P. abietis* appears to be consistently associated with the inner bark of *Abies alba* in temperate regions of Europe, suggesting strong substrate specificity (Réblová 2013; Hernandez-Restrepo et al. 2017). The occurrence of multiple monotypic genera within Platytrachelaceae likely reflects pronounced ecological differentiation among lineages, particularly with respect to habitat type and substrate preference. Such ecological specialization may constrain species diversification within individual lineages, resulting in phylogenetically distinct but species-poor taxa. Nevertheless, given the limited number of collections currently available for most members of the family, especially microfungi from freshwater environments, expanded sampling across a broader range of habitats and geographic regions may reveal additional diversity within Platytrachelaceae.

Exploration of regional fungal diversity remains essential for understanding global mycobiota. Ongoing studies on lignicolous freshwater fungi in southwestern China have yielded several novel taxa, including *M. aquaticum*, as described herein (Luo et al. 2019; Dong et al. 2020; Bao et al. 2021; Shen et al. 2022b, 2024a; Wang et al. 2024b; Xu et al. 2025b). These findings enrich knowledge of fungal diversity in the region and contribute insights into phylogenetic relationships and distribution patterns, providing a foundation for further studies in fungal taxonomy and systematics.

## Supplementary Material

XML Treatment for Platytrachelaceae

XML Treatment for Multiseptisporium

XML Treatment for Multiseptisporium aquaticum

## Additional information

### Use of AI

No use of AI was reported.

### Author ORCIDs

Hong-Wei Shen https://orcid.org/0000-0003-2508-1970

Dan-Feng Bao https://orcid.org/0000-0002-5697-4280

Xing-Juan Xiao https://orcid.org/0009-0003-8769-4534

Jayarama Darbhe Bhat https://orcid.org/0000-0002-3800-5910

Yong-Zhong Lu https://orcid.org/0000-0002-1033-5782

Zong-Long Luo https://orcid.org/0000-0001-7307-4885

### Data availability

All of the data that support the findings of this study are available in the main text or Supplementary Information.

## Supplementary materials

Supplementary material 1 Phylogenetic trees Data type docx https://binary.pensoft.net/file/1532692This dataset is made available under the Open Database License (http://opendatacommons.org/licenses/odbl/1.0/). The Open Database License (ODbL) is a license agreement intended to allow users to freely share, modify, and use this Dataset while maintaining this same freedom for others, provided that the original source and author(s) are credited. Hong-Wei Shen, Dan-Feng Bao, Xing-Juan Xiao, Jayarama Darbhe Bhat, Yong-Zhong Lu, Zong-Long Luo

## References

[B1] Bao DF, Luo ZL, Liu JK, Bhat DJ, Sarunya N, Li WL, Su HY, Hyde KD (2018) Lignicolous freshwater fungi in China III: Three new species and a new record of *Kirschsteiniothelia* from northwestern Yunnan Province. Mycosphere : Journal of Fungal Biology 9: 755–768. 10.5943/mycosphere/9/4/4

[B2] Bao DF, Su HY, Maharachchikumbura SSN, Liu JK, Nalumpang S, Luo ZL, Hyde KD (2019) Lignicolous freshwater fungi from China and Thailand: Multi-gene phylogeny reveals new species and new records in Lophiostomataceae. Mycosphere : Journal of Fungal Biology 10: 1080–1099. 10.5943/mycosphere/10/1/20

[B3] Bao DF, McKenzie EHC, Bhat DJ, Hyde KD, Luo ZL, Shen HW, Su HY (2020) *Acrogenospora* (Acrogenosporaceae, Minutisphaerales) appears to be a very diverse genus. Frontiers in Microbiology 11: 1606. 10.3389/fmicb.2020.01606 PMC7393737 32793142

[B4] Bao DF, Hyde KD, McKenzie EHC, Jeewon R, Su HY, Nalumpang S, Luo ZL (2021) Biodiversity of lignicolous freshwater hyphomycetes from China and Thailand and description of sixteen species. Journal of Fungi (Basel, Switzerland) 7: 669. 10.3390/jof7080669 PMC8399276 34436208

[B5] Bao DF, Hyde KD, Maharachchikumbura SSN, Perera RH, Thiyagaraja V, Hongsanan S, Wanasinghe DN, Shen HW, Tian XG, Yang LQ, Nalumpang S, Luo ZL (2023) Taxonomy, phylogeny and evolution of freshwater Hypocreomycetidae (Sordariomycetes). Fungal Diversity 121: 1–94. 10.1007/s13225-023-00521-8

[B6] Bhunjun CS, Phillips AJL, Jayawardena RS, Promputtha I, Hyde KD (2021) Importance of Molecular Data to Identify Fungal Plant Pathogens and Guidelines for Pathogenicity Testing Based on Koch’s Postulates. Pathogens (Basel, Switzerland) 10: 1096. 10.3390/pathogens10091096 PMC8465164 34578129

[B7] Bucher VVC, Hyde KD, Pointing SB, Reddy CA (2004) Production of wood decay enzymes, mass loss and lignin solubilization in wood by marine ascomycetes and their anamorphs. Fungal Diversity 15: 1–14.

[B8] Cai L, Tsui CKM, Zhang KQ, Hyde KD (2002) Aquatic fungi from Lake Fuxian, Yunnan, China. Fungal Diversity 9: 57–70.

[B9] Calabon MS, Jones EBG, Boonmee S, Doilom M, Lumyong S, Hyde KD (2021) Five novel freshwater Ascomycetes indicate high undiscovered diversity in lotic habitats in Thailand. Journal of Fungi (Basel, Switzerland) 7: 117. 10.3390/jof7020117 PMC7914987 33562556

[B10] Calabon MS, Hyde KD, Jones EBG, Luo ZL, Dong W, Hurdeal VG, Gentekaki E, Rossi W, Leonardi M, Thiyagaraja V, Lestari AS, Shen HW, Bao DF, Boonyuen N, Zeng M (2022) Freshwater fungal numbers. Fungal Diversity 114: 3–235. 10.1007/s13225-022-00503-2

[B11] Calabon MS, Hyde KD, Jones EBG, Bao DF, Bhunjun CS, Phukhamsakda C, Shen HW, Gentekaki E, Al Sharie AH, Barros J, Chandrasiri KSU, Hu DM, Hurdeal VG, Rossi W, Valle LG, Zhang H, Figueroa M, Raja HA, Seena S, Song HY, Dong W, El-Elimat T, Leonardi M, Li Y, Li YJ, Luo ZL, Ritter CD, Strongman DB, Wei MJ, Balasuriya A (2023) Freshwater fungal biology. Mycosphere: Journal of Fungal Biology 14: 195–413. 10.5943/mycosphere/14/1/4

[B12] Capella-Gutiérrez S, Silla-Martinez JM, Gabaldon T (2009) trimAl: A tool for automated alignment trimming in large-scale phylogenetic analyses. Bioinformatics (Oxford, England) 25: 1972–1973. 10.1093/bioinformatics/btp348 PMC2712344 19505945

[B13] Chen YP, Su PW, Hyde KD, Maharachchikumbura SSN (2023) Phylogenomics and diversification of Sordariomycetes. Mycosphere: Journal of Fungal Biology 14: 414–451. 10.5943/mycosphere/14/1/5

[B14] Crous PW, Groenewald JZ (2013) A phylogenetic re-evaluation of *Arthrinium*. IMA Fungus 4: 133–154. 10.5598/imafungus.2013.04.01.13 PMC3719201 23898419

[B15] Crous PW, Carnegie AJ, Wingfield MJ, Sharma R, Mughini G, Noordeloos ME, Santini A, Shouche YS, Bezerra JDP, Dima B, Guarnaccia V, Imrefi I, Jurjevic Z, Knapp DG, Kovacs GM, Magista D, Perrone G, Rama T, Rebriev YA, Shivas RG, Singh SM, Souza-Motta CM, Thangavel R, Adhapure NN, Alexandrova AV, Alfenas AC, Alfenas RF, Alvarado P, Alves AL, Andrade DA, Andrade JP, Barbosa RN, Barili A, Barnes CW, Baseia IG, Bellanger JM, Berlanas C, Bessette AE, Bessette AR, Biketova AY, Bomfim FS, Brandrud TE, Bransgrove K, Brito ACQ, Cano-Lira JF, Cantillo T, Cavalcanti AD, Cheewangkoon R, Chikowski RS, Conforto C, Cordeiro TRL, Craine JD, Cruz R, Damm U, de Oliveira RJV, de Souza JT, de Souza HG, Dearnaley JDW, Dimitrov RA, Dovana F, Erhard A, Esteve-Raventos F, Felix CR, Ferisin G, Fernandes RA, Ferreira RJ, Ferro LO, Figueiredo CN, Frank JL, Freire K, Garcia D, Gene J, Gesiorska A, Gibertoni TB, Gondra RAG, Gouliamova DE, Gramaje D, Guard F, Gusmao LFP, Haitook S, Hirooka Y, Houbraken J, Hubka V, Inamdar A, Iturriaga T, Iturrieta-Gonzalez I, Jadan M, Jiang N, Justo A, Kachalkin AV, Kapitonov VI, Karadelev M, Karakehian J, Kasuya T, Kautmanova I, Kruse J, Kusan I, Kuznetsova TA, Landell MF, Larsson KH, Lee HB, Lima DX, Lira CRS, Machado AR, Madrid H, Magalhaes OMC, Majerova H, Malysheva EF, Mapperson RR, Marbach PAS, Martin MP, Martin-Sanz A, Matocec N, McTaggart AR, Mello JF, Melo RFR, Mesic A, Michereff SJ, Miller AN, Minoshima A, Molinero-Ruiz L, Morozova OV, Mosoh D, Nabe M, Naik R, Nara K, Nascimento SS, Neves RP, Olariaga I, Oliveira RL, Oliveira TGL, Ono T, Ordonez ME, Ottoni AM, Paiva LM, Pancorbo F, Pant B, Pawlowska J, Peterson SW, Raudabaugh DB, Rodriguez-Andrade E, Rubio E, Rusevska K, Santiago A, Santos ACS, Santos C, Sazanova NA, Shah S, Sharma J, Silva BDB, Siquier JL, Sonawane MS, Stchigel AM, Svetasheva T, Tamakeaw N, Telleria MT, Tiago PV, Tian CM, Tkalcec Z, Tomashevskaya MA, Truong HH, Vecherskii MV, Visagie CM, Vizzini A, Yilmaz N, Zmitrovich IV, Zvyagina EA, Boekhout T, Kehlet T, Laessoe T, Groenewald JZ (2019) Fungal Planet description sheets: 868–950. Persoonia 42: 291–473. 10.3767/persoonia.2019.42.11 PMC6712538 31551622

[B16] Crous PW, Lombard L, Sandoval-Denis M, Seifert KA, Schroers HJ, Chaverri P, Gené J, Guarro J, Hirooka Y, Bensch K, Kema GHJ, Lamprecht SC, Cai L, Rossman AY, Stadler M, Summerbell RC, Taylor JW, Ploch S, Visagie CM, Yilmaz N, Frisvad JC, Abdel-Azeem AM, Abdollahzadeh J, Abdolrasouli A, Akulov A, Alberts JF, Araújo JPM, Ariyawansa HA, Bakhshi M, Bendiksby M, Ben Hadj Amor A, Bezerra JDP, Boekhout T, Câmara MPS, Carbia M, Cardinali G, Castañeda-Ruiz RF, Celis A, Chaturvedi V, Collemare J, Croll D, Damm U, Decock CA, de Vries RP, Ezekiel CN, Fan XL, Fernández NB, Gaya E, González CD, Gramaje D, Groenewald JZ, Grube M, Guevara-Suarez M, Gupta VK, Guarnaccia V, Haddaji A, Hagen F, Haelewaters D, Hansen K, Hashimoto A, Hernández-Restrepo M, Houbraken J, Hubka V, Hyde KD, Iturriaga T, Jeewon R, Johnston PR, Jurjević Ž, Karalti I, Korsten L, Kuramae EE, Kušan I, Labuda R, Lawrence DP, Lee HB, Lechat C, Li HY, Litovka YA, Maharachchikumbura SSN, Marin-Felix Y, Matio Kemkuignou B, Matočec N, McTaggart AR, Mlčoch P, Mugnai L, Nakashima C, Nilsson RH, Noumeur SR, Pavlov IN, Peralta MP, Phillips AJL, Pitt JI, Polizzi G, Quaedvlieg W, Rajeshkumar KC, Restrepo S, Rhaiem A, Robert J, Robert V, Rodrigues AM, Salgado-Salazar C, Samson RA, Santos ACS, Shivas RG, Souza-Motta CM, Sun GY, Swart WJ, Szoke S, Tan YP, Taylor JE, Taylor PWJ, Tiago PV, Váczy KZ, van de Wiele N, van der Merwe NA, Verkley GJM, Vieira WAS, Vizzini A, Weir BS, Wijayawardene NN, Xia JW, Yáñez-Morales MJ, Yurkov A, Zamora JC, Zare R, Zhang CL, Thines M (2021) *Fusarium*: More than a node or a foot-shaped basal cell. Studies in Mycology 98: 100116. 10.1016/j.simyco.2021.100116 PMC8379525 34466168

[B17] Darriba D, Taboada GL, Doallo R, Posada D (2012) jModelTest 2: More models, new heuristics and parallel computing. Nature Methods 9: 772. 10.1038/nmeth.2109 PMC4594756 22847109

[B18] Delgado G, Koukol O, Maciá-Vicente JG, Colbert W, Piepenbring M (2024) Redefining *Ellisembia**sensu stricto* with a reassessment of related taxa in Sordariomycetes. Mycological Progress 23: 32. 10.1007/s11557-024-01967-z

[B19] Dissanayake LS, Samarakoon MC, Mortimer PE, Lu Y-Z, Li QR, Hyde KD, Kang JC (2020) Morpho-molecular characterization of two novel amphisphaeriaceous species from Yunnan, China. Phytotaxa 446: 144–158. 10.11646/phytotaxa.446.3.1

[B20] Dissanayake AJ, Zhu JT, Chen YY, Maharachchikumbura SSN, Hyde KD, Liu JK (2024) A re-evaluation of *Diaporthe*: Refining the boundaries of species and species complexes. Fungal Diversity 126: 1–125. 10.1007/s13225-024-00538-7

[B21] Dong W, Wang B, Hyde KD, McKenzie EHC, Raja HA, Tanaka K, Abdel-Wahab MA, Abdel-Aziz FA, Doilom M, Phookamsak R, Hongsanan S, Wanasinghe DN, Yu XD, Wang G-N, Yang H, Yang J, Thambugala KM, Tian Q, Luo ZL, Yang J-B, Miller AN, Fournier J, Boonmee S, Hu DM, Nalumpang S, Zhang H (2020) Freshwater Dothideomycetes. Fungal Diversity 105: 319–575. 10.1007/s13225-020-00463-5

[B22] Dong W, Hyde KD, Jeewon R, Doilom M, Yu XD, Wang GN, Liu NG, Hu DM, Nalumpang S, Zhang H (2021a) Towards a natural classification of annulatascaceae-like taxa II: Introducing five new genera and eighteen new species from freshwater. Mycosphere : Journal of Fungal Biology 12: 1–88. 10.5943/mycosphere/12/1/1

[B23] Dong W, Jeewon R, Hyde KD, Yang EF, Zhang H, Yu XD, Wang GN, Suwannarach N, Doilom M, Dong Z (2021b) Five novel taxa from freshwater habitats and new taxonomic insights of Pleurotheciales and Savoryellomycetidae. Journal of Fungi (Basel, Switzerland) 7: 711. 10.3390/jof7090711 PMC8470061 34575749

[B24] Du HZ, Yang J, Liu NG, Cheewangkoon R, Liu JK (2022) Morpho-Phylogenetic evidence reveals new species of Fuscosporellaceae and Savoryellaceae from freshwater habitats in Guizhou Province, China. Journal of Fungi (Basel, Switzerland) 8: 1138. 10.3390/jof8111138 PMC9696266 36354905

[B25] Fryar SC, Catcheside DEA (2023) *Neospadicoides australiensis* (Xenospadicoidaceae), a new freshwater fungus from South Australia. Australian Journal of Taxonomy 25: 1–6. 10.54102/ajt.zvww7

[B26] Fryar SC, Reblova M, Catcheside DE (2023) Freshwater fungi from southern Australia: *Minivolcanus unicellularis* gen. et. sp. nov. and *Achrochaeta rivulata* sp. nov. Australian Journal of Taxonomy 40: 1–19. 10.54102/ajt

[B27] Guindon S, Gascuel O (2003) A simple, fast, and accurate algorithm to estimate large phylogenies by maximum likelihood. Systematic Biology 52: 696–704. 10.1080/10635150390235520 14530136

[B28] Hernández-Restrepo M, Silvera-Simón C, Mena-Portales J, Mercado-Sierra Á, Guarro J, Gené J (2011) Three new species and a new record of *Diplococcium* from plant debris in Spain. Mycological Progress 11: 191–199. 10.1007/s11557-011-0741-6

[B29] Hernandez-Restrepo M, Gene J, Castaneda-Ruiz RF, Mena-Portales J, Crous PW, Guarro J (2017) Phylogeny of saprobic microfungi from Southern Europe. Studies in Mycology 86: 53–97. 10.1016/j.simyco.2017.05.002 PMC5470572 28626275

[B30] Huang SK, Hyde KD, Mapook A, Maharachchikumbura SSN, Bhat JD, McKenzie EHC, Jeewon R, Wen TC (2021) Taxonomic studies of some often over-looked Diaporthomycetidae and Sordariomycetidae. Fungal Diversity 111: 443–572. 10.1007/s13225-021-00488-4

[B31] Hyde KD, Fryar S, Tian Q, Bahkali AH, Xu J (2016) Lignicolous freshwater fungi along a north–south latitudinal gradient in the Asian/Australian region; can we predict the impact of global warming on biodiversity and function? Fungal Ecology 19: 190–200. 10.1016/j.funeco.2015.07.002

[B32] Hyde KD, Norphanphoun C, Maharachchikumbura SSN, Bhat DJ, Jones EBG, Bundhun D, Chen YJ, Bao DF, Boonmee S, Calabon MS, Chaiwan N, Chethana KWT, Dai DQ, Dayarathne MC, Devadatha B, Dissanayake AJ, Dissanayake LS, Doilom M, Dong W, Fan XL, Goonasekara ID, Hongsanan S, Huang SK, Jayawardena RS, Jeewon R, Karunarathna A, Konta S, Kumar V, Lin CG, Liu JK, Liu NG, Luangsa-ard J, Lumyong S, Luo ZL, Marasinghe DS, McKenzie EHC, Niego AGT, Niranjan M, Perera RH, Phukhamsakda C, Rathnayaka AR, Samarakoon MC, Samarakoon SMBC, Sarma V, Senanayake IC, Shang QJ, Stadler M, Tibpromma S, Wanasinghe DN, Wei DP, Wijayawardene NN, Xiao YP, Yang J, Zeng XY, Zhang SN, Xiang MM (2020) Refined families of Sordariomycetes. Mycosphere : Journal of Fungal Biology 11: 305–1059. 10.5943/mycosphere/11/1/7

[B33] Hyde KD, Bao DF, Hongsanan S, Chethana KWT, Yang J, Suwannarach N (2021) Evolution of freshwater Diaporthomycetidae (Sordariomycetes) provides evidence for five new orders and six new families. Fungal Diversity 107: 71–105. 10.1007/s13225-021-00469-7

[B34] Hyde KD, Noorabadi MT, Thiyagaraja V, He MQ, Johnston PR, Wijesinghe SN, Armand A, Biketova AY, Chethana KWT, Erdoğdu M, Ge ZW, Groenewald JZ, Hongsanan S, Kušan I, Leontyev DV, Li DW, Lin CG, Liu NG, Maharachchikumbura SSN, Matočec N, May TW, McKenzie EHC, Mešić A, Perera RH, Phukhamsakda C, Piątek M, Samarakoon MC, Selcuk F, Senanayake IC, Tanney JB, Tian Q, Vizzini A, Wanasinghe DN, Wannasawang N, Wijayawardene NN, Zhao RL, Abdel-Wahab MA, Abdollahzadeh J, Abeywickrama PD, Abhinav Absalan S, Acharya K, Afshari N, Afshan NS, Afzalinia S, Ahmadpour SA, Akulov O, Alizadeh A, Alizadeh M, Al-Sadi AM, Alves A, Alves VCS, Alves-Silva G, Antonín V, Aouali S, Aptroot A, Apurillo CCS, Arias RM, Asgari B, Asghari R, Assis DMA, Assyov B, Atienza V, Aumentado HDR, Avasthi S, Azevedo E, Bakhshi M, Bao DF, Baral HO, Barata M, Barbosa KD, Barbosa RN, Barbosa FR, Baroncelli R, Barreto GG, Baschien C, Bennett RM, Bera I, Bezerra JDP, Bhunjun CS, Bianchinotti MV, Błaszkowski J, Boekhout T, Bonito GM, Boonmee S, Boonyuen N, Bortnikov FM, Bregant C, Bundhun D, Burgaud G, Buyck B, Caeiro MF, Cabarroi-Hernández M, Cai MF, Cai L, Calabon MS, Calaça FJS, Callalli M, Câmara MPS, Cano-Lira J, Cao B, Carlavilla JR, Carvalho A, Carvalho TG, Castañeda-Ruiz RF, Catania MDV, Cazabonne J, Cedeño-Sanchez M, Chaharmiri-Dokhaharani S, Chaiwan N, Chakraborty N, Cheewankoon R, Chen C, Chen J, Chen Q, Chen YP, Chinaglia S, Coelho-Nascimento CC, Coleine C, Costa-Rezende DH, Cortés-Pérez A, Crouch JA, Crous PW, Cruz RHSF, Czachura P, Damm U, Darmostuk V, Daroodi Z, Das K, Das K, Davoodian N, Davydov EA, da Silva GA, da Silva IR, da Silva RMF, da Silva Santos AC, Dai DQ, Dai YC, de Groot MD, De Kesel A, De Lange R, de Medeiros EV, de Souza CFA, de Souza FA, dela Cruz TEE, Decock C, Delgado G, Denchev CM, Denchev TT, Deng YL, Dentinger BTM, Devadatha B, Dianese JC, Dima B, Doilom M, Dissanayake AJ, Dissanayake DMLS, Dissanayake LS, Diniz AG, Dolatabadi S, Dong JH, Dong W, Dong ZY, Drechsler-Santos ER, Druzhinina IS, Du TY, Dubey MK, Dutta AK, Elliott TF, Elshahed MS, Egidi E, Eisvand P, Fan L, Fan X, Fan XL, Fedosova AG, Ferro LO, Fiuza PO, Flakus A, Fonseca EO, Fryar SC, Gabaldón T, Gajanayake AJ, Gannibal PB, Gao F, García-Sánchez D, García-Sandoval R, Garrido-Benavent I, Garzoli L, Gasca-Pineda J, Gautam AK, Gené J, Ghobad NM, Ghosh A, Giachini AJ, Gibertoni TB, Gentekaki E, Gmoshinskiy VI, Góes-Neto A, Gomdola D, Gorjón SP, Goto BT, Granados-Montero MM, Griffith GW, Groenewald M, Grossart H-P, Gu ZR, Gueidan C, Gunarathne A, Gunaseelan S, Guo SL, Gusmão LFP, Gutierrez AC, Guzmán-Dávalos L, Haelewaters D, Haituk H, Halling RE, He SC, Heredia G, Hernández-Restrepo M, Hosoya T, Hoog SD, Horak E, Hou CL, Houbraken J, Htet ZH, Huang SK, Huang WJ, Hurdeal VG, Hustad VP, Inácio CA, Janik P, Jayalal RGU, Jayasiri SC, Jayawardena RS, Jeewon R, Jerônimo GH, Jin J, Jones EBG, Joshi Y, Jurjević Ž, Justo A, Kakishima M, Kaliyaperumal M, Kang GP, Kang JC, Karimi O, Karunarathna SC, Karpov SA, Kezo K, Khalid AN, Khan MK, Khuna S, Khyaju S, Kirchmair M, Klawonn I, Kraisitudomsook N, Kukwa M, Kularathnage ND, Kumar S, Lachance MA, Lado C, Latha KPD, Lee HB, Leonardi M, Lestari AS, Li C, Li H, Li J, Li Q, Li Y, Li YC, Li YX, Liao CF, Lima JLR, Lima JMS, Lima NB, Lin L, Linaldeddu BT, Linn MM, Liu F, Liu JK, Liu JW, Liu S, Liu SL, Liu XF, Liu XY, Longcore JE, Luangharn T, Luangsa-ard JJ, Lu L, Lu YZ, Lumbsch HT, Luo L, Luo M, Luo ZL, Ma J, Madagammana AD, Madhushan A, Madrid H, Magurno F, Magyar D, Mahadevakumar S, Malosso E, Malysh JM, Mamarabadi M, Manawasinghe IS, Manfrino RG, Manimohan P, Mao N, Mapook A, Marchese P, Marasinghe DS, Mardones M, MarinFelix, Y, Masigol H, Mehrabi M, Mehrabi-Koushki M, Meiras-Ottoni A, de Melo RFR, Mendes-Alvarenga RL, Mendieta S, Meng QF, Menkis A, Menolli Jr N, Mikšík M, Miller SL, Moncada B, Moncalvo JM, Monteiro JS, Monteiro M, Mora-Montes HM, Moroz EL, Moura JC, Muhammad U, Mukhopadhyay S, Nagy GL, Najam ul Sehar A, Najafiniya M, Nanayakkara CM, Naseer A, Nascimento ECR, Nascimento SS, Neuhauser S, Neves MA, Niazi AR, Nie Y, Nilsson RH, Nogueira PTS, Novozhilov YK, Noordeloos M, Norphanphoun C, Nuñez Otaño N, O’Donnell RP, Oehl F, Oliveira JA, Oliveira Junior I, Oliveira NVL, Oliveira PHF, Orihara T, Oset M, Pang KL, Papp V, Pathirana LS, Peintner U, Pem D, Pereira OL, Pérez-Moreno J, Pérez-Ortega S, Péter G, Pires-Zottarelli CLA, Phonemany M, Phongeun S, Pošta A, Prazeres JFSA, Quan Y, Quandt CA, Queiroz MB, Radek R, Rahnama K, Raj KNA, Rajeshkumar KC, Rajwar S, Ralaiveloarisoa AB, Rämä T, Ramírez-Cruz V, Rambold G, Rathnayaka AR, Raza M, Ren GC, Rinaldi AC, Rivas-Ferreiro M, Robledo GL, Ronikier A, Rossi W, Rusevska K, Ryberg M, Safi A, Salimi F, Salvador-Montoya CA, Samant B, Samaradiwakara NP, Sánchez-Castro I, Sandoval-Denis M, Santiago ALCMA, Santos ACDS, Santos LA, Sarma VV, Sarwar S, Savchenko A, Savchenko K, Saxena RK, Schoutteten N, Selbmann L, Ševčíková H, Sharma A, Shen HW, Shen YM, Shu YX, Silva HF, Silva-Filho AGS, Silva VSH, Simmons DR, Singh R, Sir EB, Sohrabi M, Souza FA, Souza-Motta CM, Sriindrasutdhi V, Sruthi OP, Stadler M, Stemler J, Stephenson SL, Stoyneva-Gaertner MP, Strassert JFH, Stryjak-Bogacka M, Su H, Sun YR, Svantesson S, Sysouphanthong P, Takamatsu S, Tan TH, Tanaka K, Tang C, Tang X, Taylor JE, Taylor PWJ, Tennakoon DS, Thakshila SAD, Thambugala KM, Thamodini GK, Thilanga D, Thines M, Tiago PV, Tian XG, Tian WH, Tibpromma S, Tkalčec Z, Tokarev YS, Tomšovský M, Torruella G, Tsurykau A, Udayanga D, Ulukapı M, Untereiner WA, Usman M, Uzunov BA, Vadthanarat S, Valenzuela R, Van den Wyngaert S, Van Vooren N, Velez P, Verma RK, Vieira LC, Vieira WAS, Vinzelj JM, Tang AMC, Walker A, Walker AK, Wang QM, Wang Y, Wang XY, Wang ZY, Wannathes N, Wartchow F, Weerakoon G, Wei DP, Wei X, White JF, Wijesundara DSA, Wisitrassameewong K, Worobiec G, Wu HX, Wu N, Xiong YR, Xu B, Xu JP, Xu R, Xu RF, Xu RJ, Yadav S, Yakovchenko LS, Yang HD, Yang X, Yang YH, Yang Y, Yang YY, Yoshioka R, Youssef NH, Yu FM, Yu ZF, Yuan LL, Yuan Q, Zabin DA, Zamora JC, Zapata CV, Zare R, Zeng M, Zeng XY, Zhang JF, Zhang JY, Zhang S, Zhang XC, Zhao CL, Zhao H, Zhao Q, Zhao H, Zhao HJ, Zhou HM, Zhu XY, Zmitrovich IV & Zucconi L & Zvyagina E (2024) The 2024 Outline of Fungi and fungus-like taxa. Mycosphere 15: 5146–6239. 10.5943/mycosphere/15/1/25

[B35] Jayawardena RS, Bhunjun CS, Hyde KD, Gentekaki E, Itthayakorn P (2021) *Colletotrichum*: Lifestyles, biology, morpho-species, species complexes and accepted species. Mycosphere: Journal of Fungal Biology 12: 519–669. 10.5943/mycosphere/12/1/7

[B36] Jones EBG, Hyde KD, Pang KL (2014) Freshwater fungi and fungi-like Organisms. De Gruyter, Berlin. 10.1515/9783110333480

[B37] Katoh K, Rozewicki J, Yamada KD (2019) MAFFT online service: Multiple sequence alignment, interactive sequence choice and visualization. Briefings in Bioinformatics 20: 1160–1166. 10.1093/bib/bbx108 PMC6781576 28968734

[B38] Koukol O, Delgado G (2021) Why morphology matters: The negative consequences of hasty descriptions of putative novelties in asexual ascomycetes. IMA Fungus 12: 26. 10.1186/s43008-021-00073-z PMC8459516 34551825

[B39] Kuraku S, Zmasek CM, Nishimura O, Katoh K (2013) aLeaves facilitates on-demand exploration of metazoan gene family trees on MAFFT sequence alignment server with enhanced interactivity. Nucleic Acids Research 41: W22–W28. 10.1093/nar/gkt389 PMC3692103 23677614

[B40] Li CX, Yu XD, Dong W, Hu DM, Boonmee S, Zhang H (2021) Freshwater hyphomycetes in Sordariomycetes: Two new species of *Tainosphaeria* (Chaetosphaeriaceae, Chaetosphaeriales) from Thailand. Phytotaxa 509: 56–68. 10.11646/phytotaxa.509.1.2

[B41] Liao CF, Doilom M, Jeewon R, Hyde KD, Manawasinghe IS, Chethana KWT, Balasuriya A, Thakshila SAD, Luo M, Mapook A, Htet ZH, Koodalugodaarachchi V, Wijekoon N, Saxena RK, Senanayake IC, Kularathnage ND, Alrefaei AF, Dong W (2025) Challenges and update on fungal endophytes: Classification, definition, diversity, ecology, evolution and functions. Fungal Diversity 131: 301–367. 10.1007/s13225-025-00550-5

[B42] Liu YJ, Whelen S, Hall BD (1999) Phylogenetic relationships among ascomycetes evidence from an RNA polymerse II subunit. Molecular Biology and Evolution 16: 1799–1808. 10.1093/oxfordjournals.molbev.a026092 10605121

[B43] Liu NG, Hyde KD, Sun YR, Bhat DJ, Jones EBG, Jumpathong J, Lin CG, Lu YZ, Yang J, Liu LL, Liu ZY, Liu JK (2024) Notes, outline, taxonomy and phylogeny of brown-spored hyphomycetes. Fungal Diversity 129: 1–281. 10.1007/s13225-024-00539-6

[B44] Liu LL, Liu YX, Chen YY, Gou JL, Chi F, Liu Y, Gu XF, Wei QQ, Zhang M, Liu ZY, Zhou S (2025) Freshwater fungi in the karst plateau wetlands from Guizhou Province, China: Taxonomic novelties in Melanommataceae (Pleosporales). MycoKeys 113: 209–236. 10.3897/mycokeys.113.140684 PMC11829198 39959309

[B45] Luo J, Yin JF, Cai L, Zhang KQ, Hyde KD (2004) Freshwater fungi in Lake Dianchi, a heavily polluted lake in Yunnan, China. Fungal Diversity 16: 93–112.

[B46] Luo ZL, Bao DF, Bhat JD, Yang J, Chai HM, Li SH, Bahkali AH, Su HY, Hyde KD (2016) *Sporoschisma* from submerged wood in Yunnan, China. Mycological Progress 15: 1145–1155. 10.1007/s11557-016-1236-2

[B47] Luo ZL, Bhat DJ, Jeewon R, Boonmee S, Bao DF, Zhao YC, Chai H-M, Su HY, Su XJ, Hyde KD (2017) Molecular phylogeny and morphological characterization of asexual fungi (Tubeufiaceae) from freshwater habitats in Yunnan, China. Cryptogamie. Mycologie 38: 27–53. 10.7872/crym/v38.iss1.2017.27

[B48] Luo ZL, Hyde KD, Liu JK, Bhat DJ, Bao DF, Li WL, Su HY (2018a) Lignicolous freshwater fungi from China II: Novel *Distoseptispora* (Distoseptisporaceae) species from northwestern Yunnan Province and a suggested unified method for studying lignicolous freshwater fungi. Mycosphere: Journal of Fungal Biology 9: 444–461. 10.5943/mycosphere/9/3/2

[B49] Luo ZL, Hyde KD, Bhat DJ, Jeewon R, Maharachchikumbura SSN, Bao DF, Li WL, Su XJ, Yang XY, Su HY (2018b) Morphological and molecular taxonomy of novel species Pleurotheciaceae from freshwater habitats in Yunnan, China. Mycological Progress 17: 511–530. 10.1007/s11557-018-1377-6

[B50] Luo ZL, Hyde KD, Liu JK, Maharachchikumbura SSN, Jeewon R, Bao D-F, Bhat DJ, Lin CG, Li WL, Yang J, Liu NG, Lu Y-Z, Jayawardena RS, Li JF, Su HY (2019) Freshwater Sordariomycetes. Fungal Diversity 99: 451–660. 10.1007/s13225-019-00438-1

[B51] Ma J, Hyde KD, Tibpromma S, Gomdola D, Liu N-G, Norphanphoun C, Bao DF, Boonmee S, Xiao XJ, Zhang LJ, Luo ZL, Zhao Q, Suwannarach N, Karunarathna SC, Liu JK, Lu YZ (2024) Taxonomy and systematics of lignicolous helicosporous hyphomycetes. Fungal Diversity 129: 365–653. 10.1007/s13225-024-00544-9

[B52] Maharachchikumbura SSN, Hyde KD, Jones EBG, McKenzie EHC, Huang SK, Abdel-Wahab MA, Daranagama DA, Dayarathne M, D’souza MJ, Goonasekara ID, Hongsanan S, Jayawardena RS, Kirk PM, Konta S, Liu JK, Liu ZY, Norphanphoun C, Pang KL, Perera RH, Senanayake IC, Shang Q, Shenoy BD, Xiao YP, Bahkali AH, Kang JC, Somrothipol S, Suetrong S, Wen TC, Xu JC (2015) Towards a natural classification and backbone tree for Sordariomycetes. Fungal Diversity 72: 199–301. 10.1007/s13225-015-0331-z

[B53] Maharachchikumbura SSN, Chen Y, Ariyawansa HA, Hyde KD, Haelewaters D, Perera RH, Samarakoon MC, Wanasinghe DN, Bustamante DE, Liu JK, Lawrence DP, Cheewangkoon R, Stadler M (2021) Integrative approaches for species delimitation in Ascomycota. Fungal Diversity 109: 155–179. 10.1007/s13225-021-00486-6

[B54] Miller MA, Pfeiffer W, Schwartz T (2010) Creating the CIPRES Science Gateway for Inference of Large Phylogenetic Trees. 2010 gateway computing environments workshop (GCE), 8 pp. 10.1109/GCE.2010.5676129

[B55] Monkai J, Phookamsak R, Tennakoon DS, Bhat DJ, Xu S, Li QX, Xu JC, Mortimer PE, Kumla J, Lumyong S (2022) Insight into the taxonomic resolution of *Apiospora*: Introducing novel species and records from bamboo in China and Thailand. Diversity 14: 918. 10.3390/d14110918

[B56] Norphanphoun C, Gentekaki E, Hongsanan S, Jayawardena R, Senanayake IC, Manawasinghe IS, Abeywickrama PD, Bhunjun CS, Hyde KD (2022) *Diaporthe*: Formalizing the species-group concept. Mycosphere : Journal of Fungal Biology 13: 752–819. 10.5943/mycosphere/13/1/9

[B57] Pintos A, Alvarado P (2021) Phylogenetic delimitation of *Apiospora* and *Arthrinium*. Fungal Systematics and Evolution 7: 197–221. 10.3114/fuse.2021.07.10 PMC8165962 34124624

[B58] Rannala B, Yang ZH (1996) Probability distribution of molecular evolutionary trees a new method of phylogenetic inference. Journal of Molecular Evolution 43: 304–311. 10.1007/BF02338839 8703097

[B59] Réblová M (2013) Two taxonomic novelties in the Sordariomycetidae: *Ceratolenta caudata* gen. et sp. nov. and *Platytrachelon abietis* gen. et comb. nov. for *Ceratosphaeria abietis*. Mycologia 105: 462–475. 10.3852/12-199 23080023

[B60] Réblová M, Nekvindová J (2023) New genera and species with chloridium-like morphotype in the Chaetosphaeriales and Vermiculariopsiellales. Studies in Mycology 106: 199–258. 10.3114/sim.2023.106.04 PMC10825751 38298574

[B61] Réblová M, Fournier J, Štěpánek V (2016) Two new lineages of aquatic ascomycetes: *Atractospora* gen. nov. and *Rubellisphaeria* gen. et sp. nov., and a sexual morph of *Myrmecridium montsegurinum* sp. nov. Mycological Progress 15: 21. 10.1007/s11557-016-1166-z

[B62] Réblová M, Miller AN, Reblova K, Stepanek V (2018) Phylogenetic classification and generic delineation of Calyptosphaeria gen. nov., *Lentomitella*, *Spadicoides* and *Torrentispora* (Sordariomycetes). Studies in Mycology 89: 1–62. 10.1016/j.simyco.2017.11.004 PMC5773705 29367793

[B63] Réblová M, Hernandez-Restrepo M, Fournier J, Nekvindová J (2020a) New insights into the systematics of Bactrodesmium and its allies and introducing new genera, species and morphological patterns in the Pleurotheciales and Savoryellales (Sordariomycetes). Studies in Mycology 95: 415–466. 10.1016/j.simyco.2020.02.002 PMC7426232 32855744

[B64] Réblová M, Nekvindová J, Fournier J, Miller AN (2020b) Delimitation, new species and teleomorph-anamorph relationships in *Codinaea*, *Dendrophoma*, *Paragaeumannomyces* and *Striatosphaeria* (Chaetosphaeriaceae). MycoKeys 74: 17–74. 10.3897/mycokeys.74.57824 PMC7588497 33149721

[B65] Réblová M, Nekvindová J, Miller AN (2021) Phylogeny and taxonomy of *Catenularia* and similar fungi with catenate conidia. MycoKeys 81: 1–44. 10.3897/mycokeys.81.67785 PMC8213683 34163305

[B66] Réblová M, Hernández-Restrepo M, Sklenář F, Nekvindová J, Réblová K, Kolařík M (2022) Consolidation of *Chloridium*: New classification into eight sections with 37 species and reinstatement of the genera *Gongromeriza* and *Psilobotrys*. Studies in Mycology 103: 87–212. 10.3114/sim.2022.103.04 PMC10277272 37342155

[B67] Ronquist F, Teslenko M, van der Mark P, Ayres DL, Darling A, Hohna S, Larget B, Liu L, Suchard MA, Huelsenbeck JP (2012) MrBayes 3.2: Efficient Bayesian phylogenetic inference and model choice across a large model space. Systematic Biology 61: 539–542. 10.1093/sysbio/sys029 PMC3329765 22357727

[B68] Shen HW, Luo ZL (2023) A preliminary study on the diversity of lignicolous freshwater fungi in plateau lakes, Yunnan. 2023 Annual Meeting of Mycological Society of China. Guiyang City, Guizhou Province, 87 pp.

[B69] Shen HW, Bao DF, Wanasinghe DN, Boonmee S, Liu JK, Luo ZL (2022a) Novel species and records of Dictyosporiaceae from freshwater habitats in China and Thailand. Journal of Fungi (Basel, Switzerland) 8: 1200. 10.3390/jof8111200 PMC9694895 36422021

[B70] Shen HW, Bao DF, Bhat DJ, Su HY, Luo ZL (2022b) Lignicolous freshwater fungi in Yunnan Province, China: An overview. Mycology 13: 119–132. 10.1080/21501203.2022.2058638 PMC9196657 35711328

[B71] Shen HW, Bao DF, Boonmee S, Su XJ, Tian XG, Hyde KD, Luo ZL (2023) Lignicolous freshwater fungi from plateau lakes in China (I): Morphological and phylogenetic analyses reveal eight species of Lentitheciaceae, including new genus, new species and new records. Journal of Fungi (Basel, Switzerland) 9: 962. 10.3390/jof9100962 PMC10607872 37888219

[B72] Shen HW, Luo ZL, Bao DF, Luan S, Bhat DJ, Boonmee S, Wang WP, Su XJ, Li YX, Al-Otibi F, Lu YZ, Yang LQ, Hyde KD (2024a) Lignicolous freshwater fungi from China IV: Morphology and phylogeny reveal new species of Pleosporales from plateau lakes in Yunnan Province, China. Mycosphere : Journal of Fungal Biology 15: 6439–6524. 10.5943/mycosphere/15/1/28

[B73] Shen HW, Bao DF, Boonmee S, Lu YZ, Su XJ, Li YX, Luo ZL (2024b) Diversity of *Distoseptispora* (Distoseptisporaceae) taxa on submerged decaying wood from the Red River in Yunnan, China. MycoKeys 102: 1–28. 10.3897/mycokeys.102.116096 PMC10862349 38356851

[B74] Shenoy BD, Jeewon R, Wu WP, Bhat DJ, Hyde KD (2006) Ribosomal and RPB2 DNA sequence analyses suggest that *Sporidesmium* and morphologically similar genera are polyphyletic. Mycological Research 110: 916–928. 10.1016/j.mycres.2006.06.004 16908125

[B75] Stamatakis A (2006) RAxML-VI-HPC: Maximum likelihood-based phylogenetic analyses with thousands of taxa and mixed models. Bioinformatics (Oxford, England) 22: 2688–2690. 10.1093/bioinformatics/btl446 16928733

[B76] Stamatakis A, Hoover P, Rougemont J (2008) A rapid bootstrap algorithm for the RAxML Web servers. Systematic Biology 57: 758–771. 10.1080/10635150802429642 18853362

[B77] Su YY, Qi YL, Cai L (2012) Induction of sporulation in plant pathogenic fungi. Mycology 3: 195–200. 10.1080/21501203.2012.719042

[B78] Su HY, Hyde KD, Maharachchikumbura SSN, Ariyawansa HA, Luo ZL, Promputtha I, Tian Q, Lin CG, Shang QJ, Zhao YC, Chai HM, Liu XY, Bahkali AH, Bhat JD, McKenzie EHC, Zhou DQ (2016) The families Distoseptisporaceae fam. nov., Kirschsteiniotheliaceae, Sporormiaceae and Torulaceae, with new species from freshwater in Yunnan Province, China. Fungal Diversity 80: 375–409. 10.1007/s13225-016-0362-0

[B79] Talhinhas P, Baroncelli R (2021) *Colletotrichum* species and complexes: Geographic distribution, host range and conservation status. Fungal Diversity 110: 109–198. 10.1007/s13225-021-00491-9

[B80] Tang X, Jeewon R, Jayawardena RS, Gomdola D, Lu YZ, Xu RJ, Alrefaei AF, Alotibi F, Hyde KD, Kang JC (2024) Additions to the genus *Kirschsteiniothelia* (Dothideomycetes): Three novel species and a new host record, based on morphology and phylogeny. MycoKeys 110: 35–66. 10.3897/mycokeys.110.133450 PMC11535726 39502522

[B81] Vaidya G, Lohman DJ, Meier R (2011) SequenceMatrix: concatenation software for the fast assembly of multi-gene datasets with character set and codon information. Cladistics 27: 171–180. 10.1111/j.1096-0031.2010.00329.x 34875773

[B82] Vilgalys R, Hester M (1990) Rapid genetic identification and mapping of enzymatically amplified ribosomal DNA from several *Cryptococcus* species. Journal of Bacteriology 172: 4238–4246. 10.1128/jb.172.8.4238-4246.1990 PMC213247 2376561

[B83] Wang F, Wang K, Cai L, Zhao MJ, Kirk PM, Fan GM, Sun QL, Li B, Wang S, Yu ZF, Han D, Ma JC, Wu LH, Yao YJ (2023) Fungal names: A comprehensive nomenclatural repository and knowledge base for fungal taxonomy. Nucleic Acids Research 51: D708–D716. 10.1093/nar/gkac926 PMC9825588 36271801

[B84] Wang WP, Bhat DJ, Yang L, Shen HW, Luo ZL (2024a) New species and records of Pleurotheciaceae from karst kandscapes in Yunnan Province, China. Journal of Fungi (Basel, Switzerland) 10: 516. 10.3390/jof10080516 PMC11355354 39194842

[B85] Wang WP, Hyde KD, Bao DF, Wanasinghe DN, Lin CG, Shen HW, Lu YZ, Zhang ZQ, Su XJ, Li YX, Al-Otibi F, Yang LQ, Luo ZL (2024b) Lignicolous freshwater fungi from karst landscapes in Yunnan Province, China. Mycosphere: Journal of Fungal Biology 15: 6525–6640. 10.5943/mycosphere/15/1/29

[B86] Wang WP, Shen HW, Bao DF, Jeewon R, Zhang ZQ, Yang LQ, Luo ZL (2025a) Biogeography and species diversity of freshwater Savoryellomycetidae (Sordariomycetes) fungi. Mycology: 1–70. 10.1080/21501203.2025.2509809 PMC13267038 42305155

[B87] Wang WP, Lin CG, Liu TX, Shen HW, Luo ZL (2025b) Additions to the family Junewangiaceae (Sordariomycetes): Novel species and new records from freshwater habitats in Southwestern China. Frontiers in Microbiology 16: 1566263. 10.3389/fmicb.2025.1566263 PMC12053288 40330731

[B88] White T, Bruns T, Lee S, Taylor J (1990) Amplification and direct sequencing of fungal ribosomal RNA genes for phylogenetics. PCR protocols: A guide to methods and applications 18: 315–322. 10.1016/B978-0-12-372180-8.50042-1

[B89] Wu WP, Diao YZ (2022) Anamorphic chaetosphaeriaceous fungi from China. Fungal Diversity 116: 1–546. 10.1007/s13225-022-00509-w

[B90] Xiao XJ, Liu NG, Ma J, Zhang LJ, Bao DF, Bai S, Al-Otibi F, Hyde KD, Lu YZ (2025) Three new asexual *Kirschsteiniothelia* species from Guizhou Province, China. MycoKeys 113: 147–168. 10.3897/mycokeys.113.139427 PMC11811712 39936083

[B91] Xu RJ, Zhu YA, Liu NG, Boonmee S, Zhou DQ, Zhao Q (2023) Taxonomy and phylogeny of hyphomycetous muriform conidial taxa from the Tibetan Plateau, China. Journal of Fungi (Basel, Switzerland) 9: 560. 10.3390/jof9050560 PMC10220608 37233273

[B92] Xu RJ, Thiyagaraja V, Li Y, Zhou DQ, Boonmee S, Zhao Q (2024) Two novel lignicolous freshwater fungi, *Conioscypha xizangensis* and *Cordana linzhiensis*, from the Tibetan Plateau, China. New Zealand Journal of Botany: 1–17. 10.1080/0028825X.2024.2336044

[B93] Xu CY, Song HY, Zhou JP, Zhai ZJ, Cui CY, Hu DM (2025a) Four new or newly recorded species from freshwater habitats in Jiangxi Province, China. Journal of Fungi (Basel, Switzerland) 11: 79. 10.3390/jof11010079 PMC11766928 39852498

[B94] Xu RJ, Hyde KD, Li J-N, Boonmee S, Liu NG, Yang J, Li Y, Bao DF, Shen HW, Zhu XT, Zhu YA, Li TS, Xu K, Yu FM, Lu JR, Lei L, Wu N, Wu D-M, Gao N, Jia P-S, He XL, Al-Otibi F, Zhou DQ, Liu J-K, Lu YZ, Luo ZL, Yang ZL, Zhao Q (2025b) Lignicolous freshwater fungi of the pan Qinghai-Xizang Plateau, China. Fungal Diversity 133: 23–234. 10.1007/s13225-025-00555-0

[B95] Yang J, Liu JK, Hyde KD, Jones EBG, Luo ZL, Liu ZY (2019) *Aquimonospora tratensis* gen. et sp. nov. (Diaporthomycetidae, Sordariomycetes), a new lineage from a freshwater habitat in Thailand. Phytotaxa 397: 146–158. 10.11646/phytotaxa.397.2.2

[B96] Yang J, Liu LL, Jones EBG, Li WL, Hyde KD, Liu ZY (2021) Morphological variety in *Distoseptispora* and introduction of six novel species. Journal of Fungi (Basel, Switzerland) 7: 945. 10.3390/jof7110945 PMC8620209 34829232

[B97] Yang J, Liu LL, Jones EBG, Hyde KD, Liu ZY, Bao DF, Liu NG, Li WL, Shen HW, Yu XD, Liu JK (2023) Freshwater fungi from karst landscapes in China and Thailand. Fungal Diversity 119: 1–212. 10.1007/s13225-023-00514-7

[B98] Yu XD, Zhang SN, Liu JK (2023) Additions to bambusicolous fungi of Savoryellaceae from Southwest China. Journal of Fungi (Basel, Switzerland) 9: 571. 10.3390/jof9050571 PMC10220963 37233282

[B99] Yu XD, Zhang SN, Liang XD, Zhu JT, Hyde KD, Liu JK (2024) Bambusicolous fungi from Southwestern China. Mycosphere : Journal of Fungal Biology 15: 5038–5145. 10.5943/mycosphere/15/1/24

[B100] Yuen TK, Hyde KD, Hodgkiss IJ (1998) Physiological growth parameters and enzyme production in tropical freshwater fungi. Material und Organismen 32: 2–16.

[B101] Zhang H, Jones GEB, Zhou D, Bahkali AH, Hyde KD (2011) Checklist of freshwater Fungi in Thailand. Cryptogamie. Mycologie 32: 199–217. 10.7872/crym.v32.iss2.2011.199

[B102] Zhang H, Zhu R, Qing Y, Yang H, Li C, Wang G, Zhang D, Ning P (2022) Polyphasic identification of *Distoseptispora* with six new species from fresh water. Journal of Fungi (Basel, Switzerland) 8: 1063. 10.3390/jof8101063 PMC9605234 36294625

