# Supplementary material for: Morpho-phylogenetic evidence uncovers new taxa of lignicolous freshwater Sordariomycetes from Yunnan and Guizhou provinces, China
Source: MycoKeys. 2026 Feb 5;128:97–124. doi: 10.3897/mycokeys.128.180553 (PMC12902769; doi:10.3897/mycokeys.128.180553)
Supplement: Supplementary material 1 — Phylogenetic trees [file mycokeys-128-097-s001.docx]

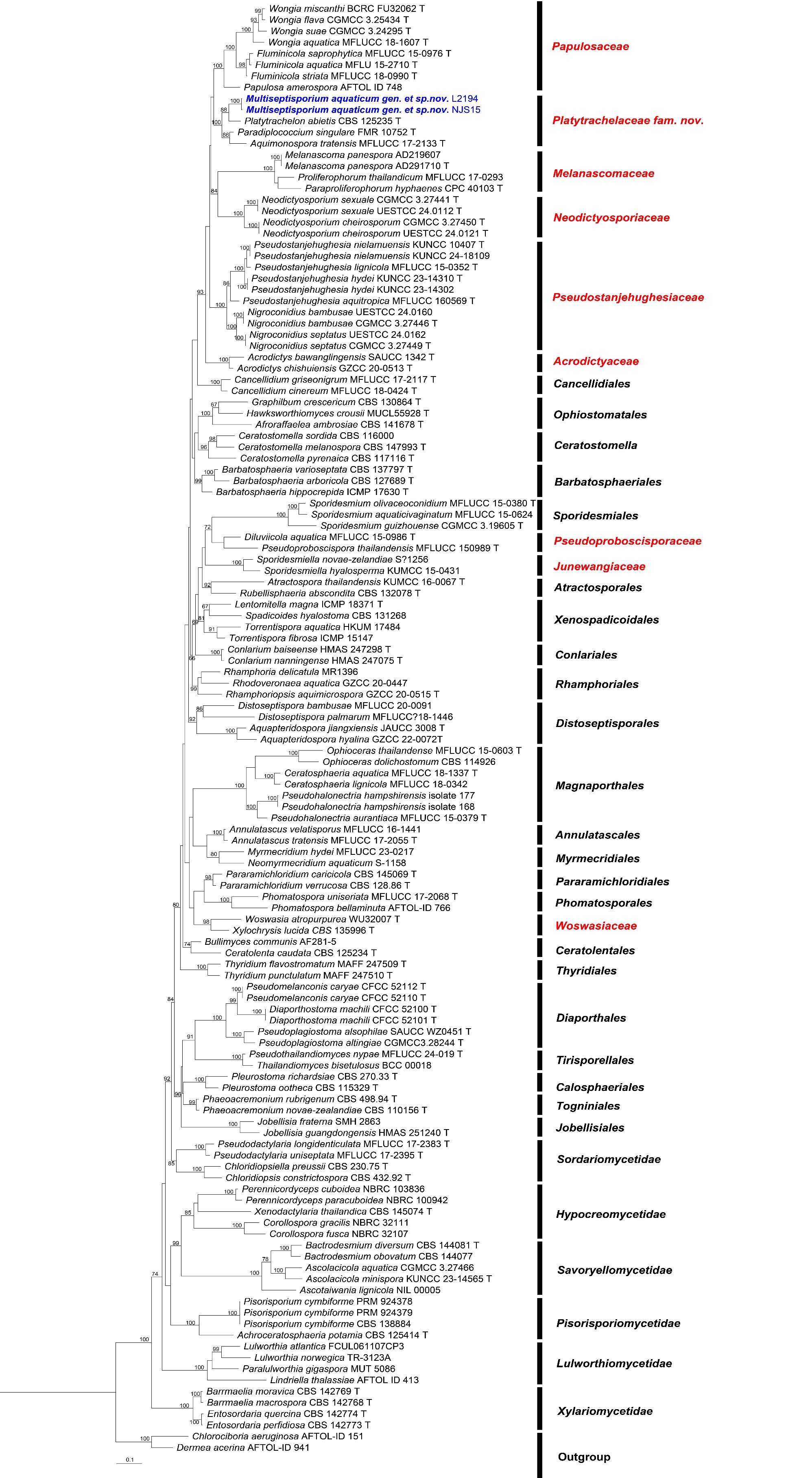


**Supplementary Figure S1** Phylogram generated from maximum likelihood analysis based on combined LSU, SSU, *rpb*2, and *tef*1-α sequences dataset representing Sordariomycetes. 129 strains are included in the combined analyses which comprise 3,565 characters including gaps (753 characters for LSU, 1,003 characters for SSU, 953 characters for *rpb*2, and 856 characters for *tef*1-α. *Chlorociboria aeruginosa* (AFTOL‑ID 151) and *Dermea acerina* (AFTOL‑ID 941) were selected as the outgroup taxa.


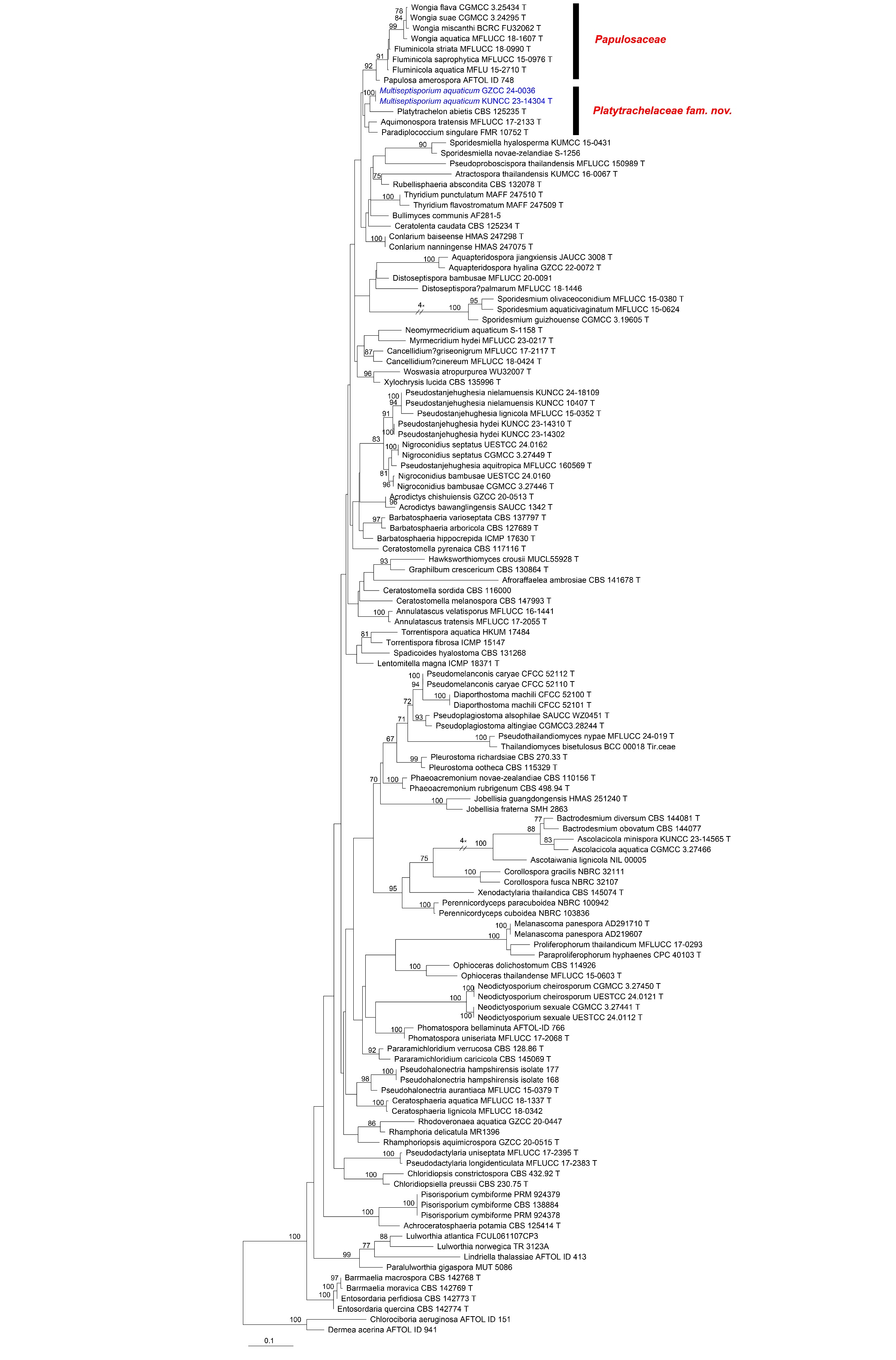


**Supplementary Figure S2** Phylogram generated from maximum likelihood analysis based on combined LSU sequences data. 128 strains are included in the combined analyses which comprise 753 characters including gaps. *Chlorociboria aeruginosa* (AFTOL‑ID 151) and *Dermea acerina* (AFTOL‑ID 941) were selected as the outgroup taxa.


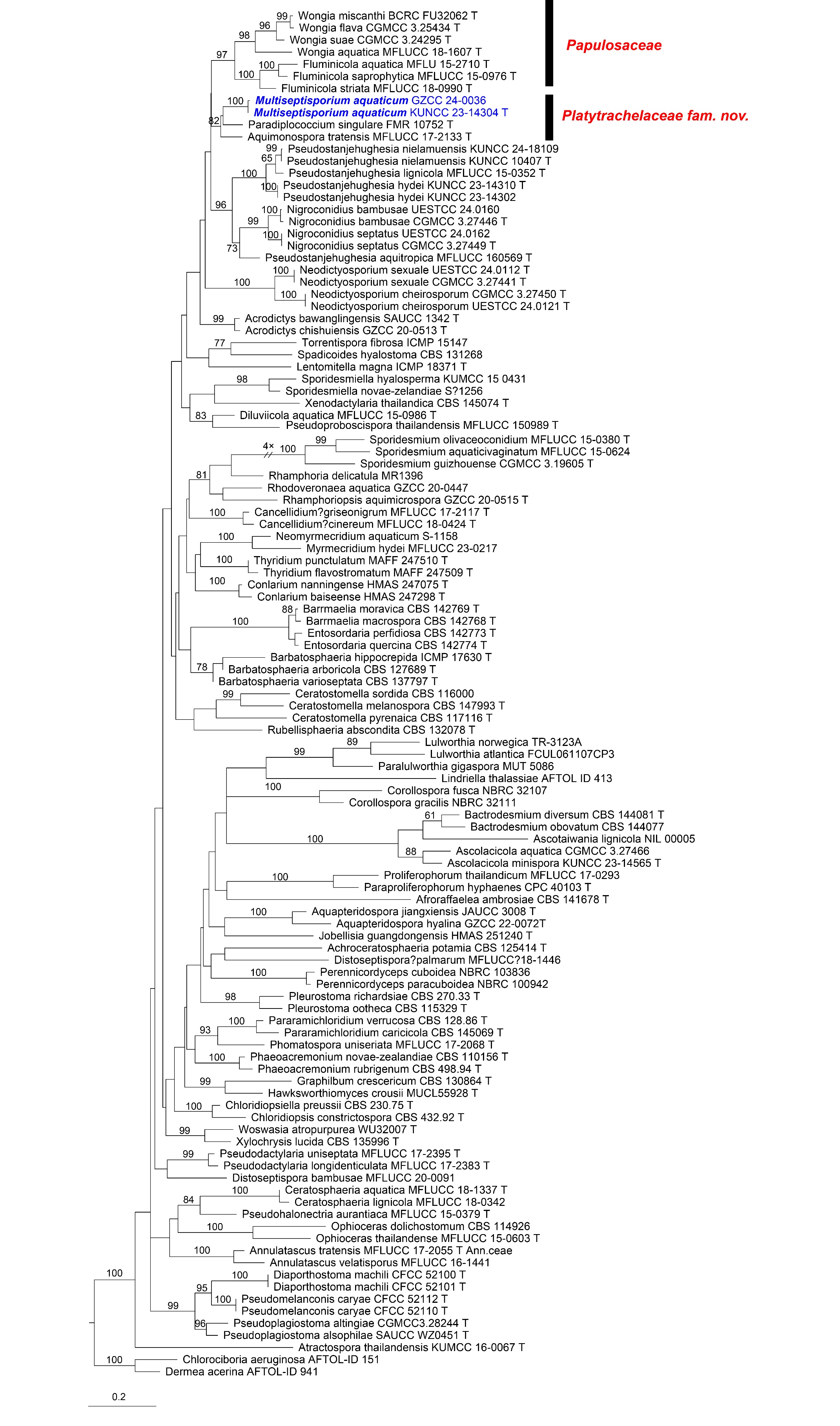


**Supplementary Figure S3** Phylogram generated from maximum likelihood analysis based on combined ITS sequences data. 113 strains are included in the combined analyses which comprise 491 characters including gaps. *Chlorociboria aeruginosa* (AFTOL‑ID 151) and *Dermea acerina* (AFTOL‑ID 941) were selected as the outgroup taxa.


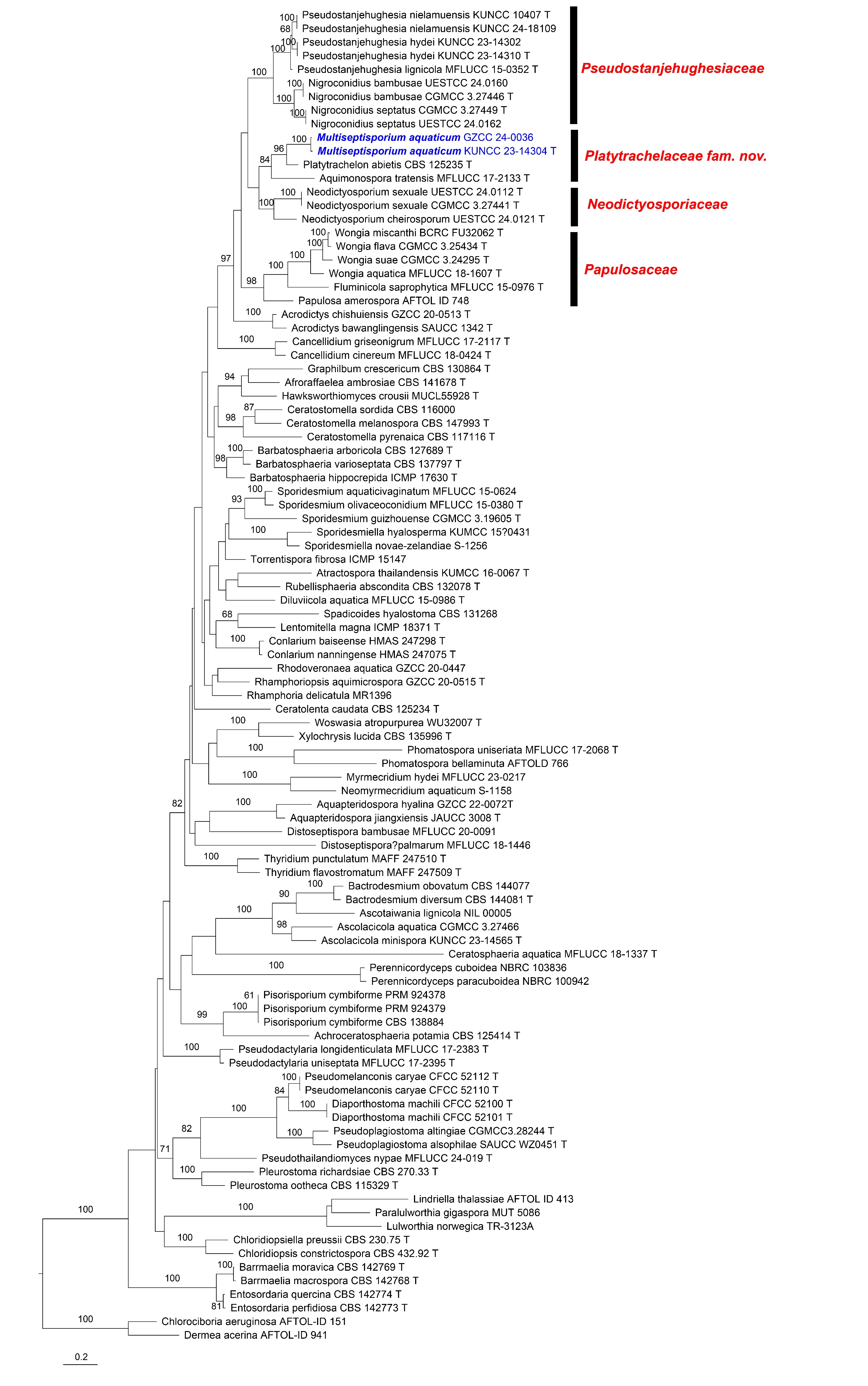


**Supplementary Figure S4** Phylogram generated from maximum likelihood analysis based on combined *rpb*2 sequences data. 98 strains are included in the combined analyses which comprise 953 characters including gaps. *Chlorociboria aeruginosa* (AFTOL‑ID 151) and *Dermea acerina* (AFTOL‑ID 941) were selected as the outgroup taxa.


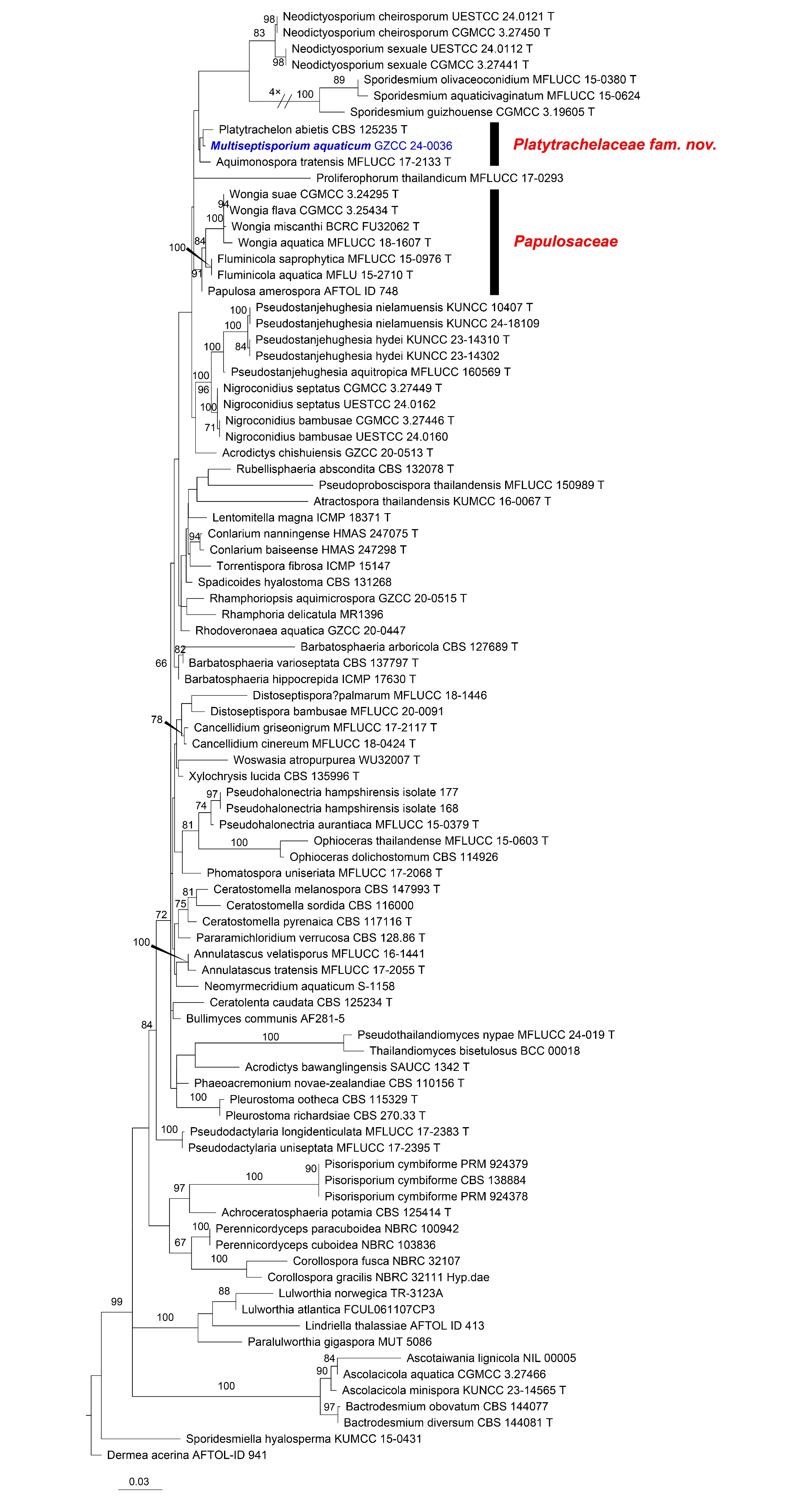


**Supplementary Figure S5** Phylogram generated from maximum likelihood analysis based on combined SSU sequences data. 90 strains are included in the combined analyses which comprise 1,003 characters including gaps. *Dermea acerina* (AFTOL‑ID 941) was selected as the outgroup taxa.


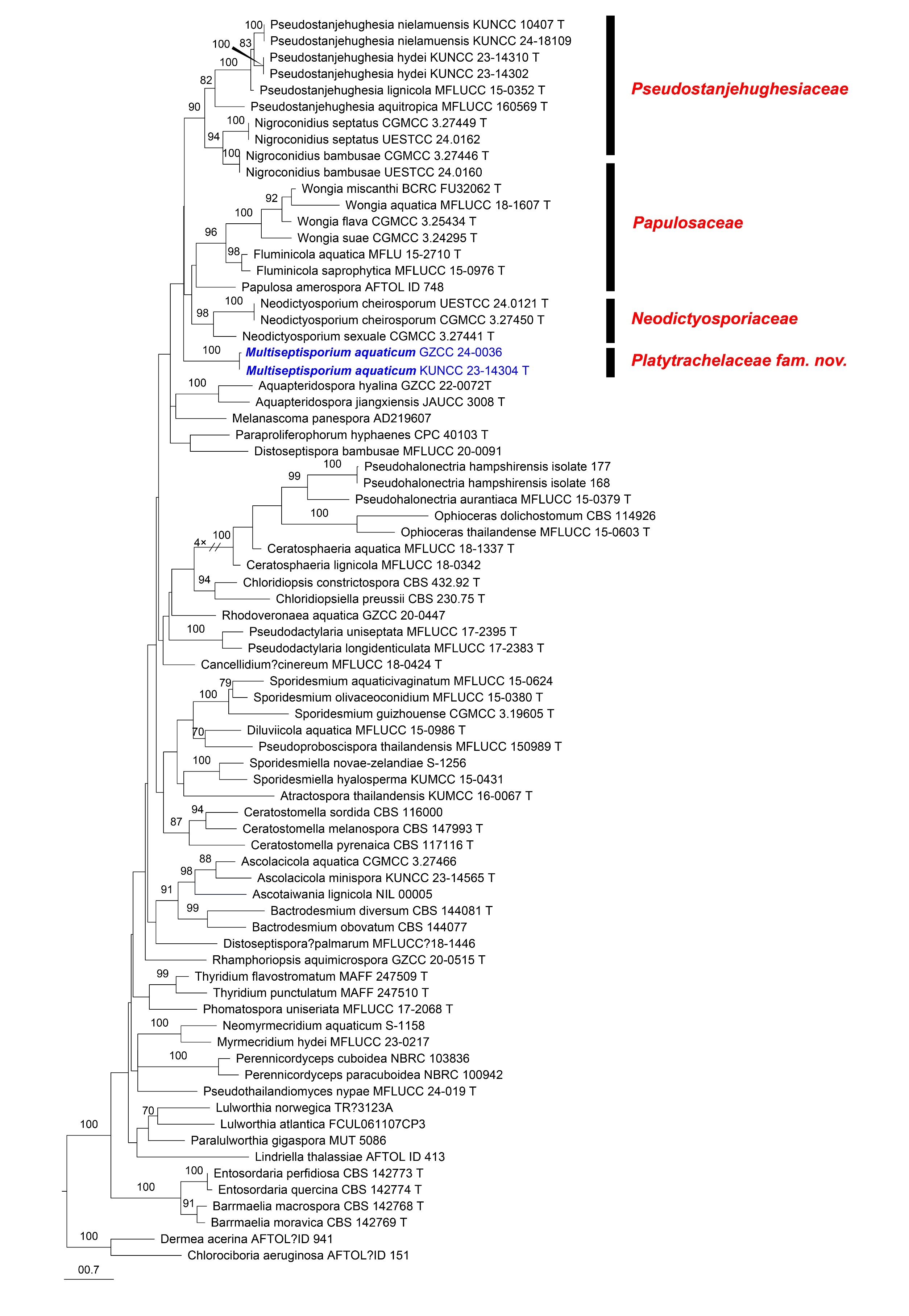


**Supplementary Figure S6** Phylogram generated from maximum likelihood analysis based on combined *tef*1-α sequences data. 76 strains are included in the combined analyses which comprise 856 characters including gaps. *Chlorociboria aeruginosa* (AFTOL‑ID 151) and *Dermea acerina* (AFTOL‑ID 941) were selected as the outgroup taxa.
